# High-Throughput High-Resolution Class I HLA Genotyping in East Africa

**DOI:** 10.1371/journal.pone.0010751

**Published:** 2010-05-20

**Authors:** Rebecca N. Koehler, Anne M. Walsh, Eric E. Sanders-Buell, Leigh Anne Eller, Michael Eller, Jeffrey R. Currier, Christian T. Bautista, Fred Wabwire-Mangen, Michael Hoelscher, Leonard Maboko, Jerome Kim, Nelson L. Michael, Merlin L. Robb, Francine E. McCutchan, Gustavo H. Kijak

**Affiliations:** 1 United States Military HIV Research Program/Henry M. Jackson Foundation, Rockville, Maryland, United States of America; 2 Makerere University Walter Reed Research Project, Henry M. Jackson Foundation, Kampala, Uganda; 3 Makerere University, Kampala, Uganda; 4 Department of Infectious Diseases and Tropical Medicine, University of Munich, Munich, Germany; 5 Mbeya Medical Research Program, Mbeya, Tanzania; 6 United States Military HIV Research Program/Walter Reed Army Institute of Research, Silver Spring, Maryland, United States of America; University of Sao Paulo, Brazil

## Abstract

HLA, the most genetically diverse loci in the human genome, play a crucial role in host-pathogen interaction by mediating innate and adaptive cellular immune responses. A vast number of infectious diseases affect East Africa, including HIV/AIDS, malaria, and tuberculosis, but the HLA genetic diversity in this region remains incompletely described. This is a major obstacle for the design and evaluation of preventive vaccines. Available HLA typing techniques, that provide the 4-digit level resolution needed to interpret immune responses, lack sufficient throughput for large immunoepidemiological studies. Here we present a novel HLA typing assay bridging the gap between high resolution and high throughput. The assay is based on real-time PCR using sequence-specific primers (SSP) and can genotype carriers of the 49 most common East African class I HLA-A, -B, and -C alleles, at the 4-digit level. Using a validation panel of 175 samples from Kampala, Uganda, previously defined by sequence-based typing, the new assay performed with 100% sensitivity and specificity. The assay was also implemented to define the HLA genetic complexity of a previously uncharacterized Tanzanian population, demonstrating its inclusion in the major East African genetic cluster. The availability of genotyping tools with this capacity will be extremely useful in the identification of correlates of immune protection and the evaluation of candidate vaccine efficacy.

## Introduction

The human leukocyte antigen (HLA) loci, located in the major histocompatibility complex (MHC), encode cell-surface molecules that present peptides sampled from the proteome, mediating key immunological events: defining self-antigen tolerance and cellular immune responses to tumors and pathogens. Class I HLA-A, -B, and -C loci are essential for both innate and adaptive cellular immune responses. Their crucial interaction with T-cell receptors on cytotoxic T-lymphocytes (CTLs) mediates adaptive immune responses against viruses and intracellular parasites [1], [2]. HLA are also ligands of killer immunoglobulin-like receptors (KIR) on the surface of natural killer cells, forming a bridge between innate and adaptive immunity [3].

The HLA are the most genetically diverse loci in the human genome [4]. When solely enumerated by variants that differ at the amino acid level (i.e., “4-digit” resolution level) the number of currently published class I HLA alleles amounts to 700 in the HLA-A locus, 1084 in the HLA-B locus, and 371 in the HLA-C locus [5]. While these counts reflect worldwide surveys, only a subset of these alleles is usually found in any given global indigenous population [6]. At the global scale, the complex genetic makeup of the HLA bears the marks of the history of each population [7], including several waves of migration [8], different levels of admixture with other populations [9], and changes in their effective population size [10]. In addition, one of the strongest forces molding HLA complexity has been the selective pressure exerted by numerous pathogens [8], [11] which is most evident in populations that have maintained larger effective population sizes for longer periods of time [12], as is the case for East African populations [13].

East African populations are heavily affected by infectious diseases [14], including malaria [15], tuberculosis [16], HIV/AIDS [17], leishmaniasis [18], schistosomiasis [19], and viral hemorrhagic fever [20], [21], [22]. For each of these diseases, HLA diversity plays a crucial role in the host-pathogen interaction, affecting the rates of disease acquisition and outcome [23], [24], [25], [26], [27], [28], [29], [30], [31]. Nevertheless, and with few exceptions [32], [33], [34], [35], [36], [37], [38], [39], [40], class I HLA genetic diversity of East African populations remains incompletely described, one of the main impediments for the design and evaluation of preventive vaccines for this region [41].

Immunoepidemiological studies aimed at supporting vaccine development require the assessment of large cohorts [42]. However, the level of diversification within HLA allele families in East African populations [32], [33] and its consequences on antigen presentation and disease course [31] call for high-resolution HLA genotyping. Currently available techniques, such as sequence-based typing (SBT), PCR using sequence-specific primers (SSP), and PCR using sequence-specific oligonucleotide probes (SSOP), meet only some of these requirements. SBT provides high-resolution typing, but at high cost and low throughput, and is not able to discern *cis*/*trans* linkage of sequence motifs, which can result in ambiguities in allele calls [43]. PCR-SSP is able to indentify linkage among polymorphisms [44], but PCR-SSP and PCR-SSOP have a lower level of resolution than SBT and require time-consuming post-PCR processing, significantly reducing their throughput.

Here we present the development, validation, and implementation of an assay to support molecular epidemiology studies capable of discriminating carriers of the most frequent class I HLA-A, -B, and -C alleles in East Africa from non-carriers, and that bridges the gap between high-throughput/low-cost and high-resolution HLA typing. The novel platform is based on real-time PCR-SSP, and performs with high sensitivity and specificity in identifying carriers of the 49 most common class I HLA-A, -B, and -C alleles in East Africa, providing 80–90% population coverage. Thus, it is an ideal tool for immunoepidemiological studies.

## Results

### Assay scope and principle

**Figure 1:**
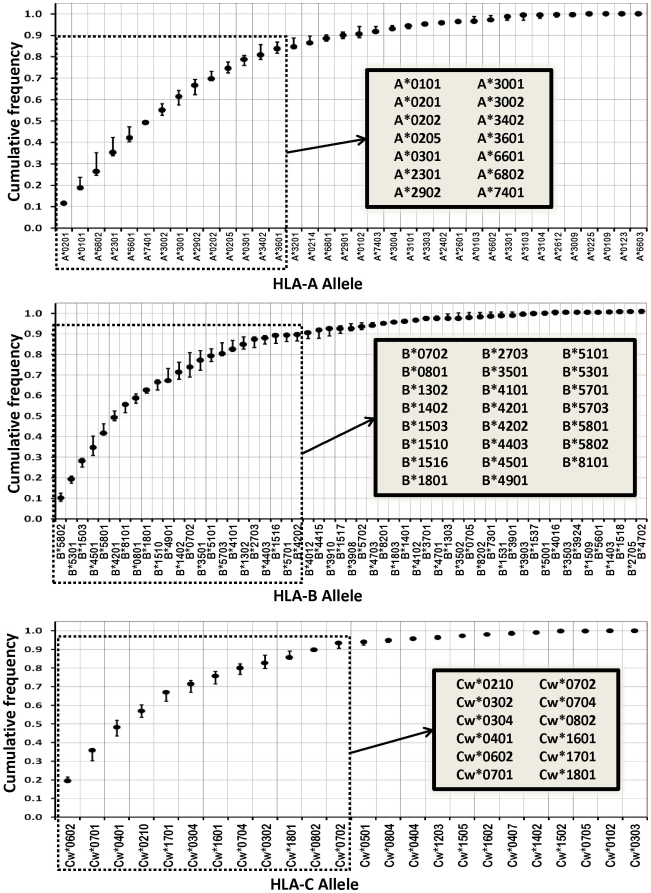
Distribution of Class I HLA-A, -B, and -C allele frequencies in East Africa. For each locus, cumulative frequencies of reported alleles in Kenyan Luo [32], Kenyan Nandi [32], and Ugandan populations [33] are depicted in decreasing frequency order. Solid dots represent the median of the cumulative frequencies among the three populations, and the error bars represent their range. HLA alleles that provide a population coverage of 80–90% and were selected as the target of the current assay (see text for details) are boxed by a dotted line and listed in the insets. Only alleles that have been reported in at least one of the three East African populations were included in the analysis.

To date, 36, 55, and 24 HLA-A, -B and -C alleles have been reported in East African populations [32], [33], respectively. There is a coincidence in the alleles constituting the major variants in Kenyan Luo, Kenyan Nandi, and Ugandans, despite some differences in the frequencies at which each allele variant is represented [32], [33]. When these alleles were sorted in descending order of abundance and the cumulative allele frequencies were calculated for each locus, their layout resembled a logarithmic distribution, with less than half of the allelic variants providing large population coverage and the remainder found at very low frequencies (Figure 1). Based on this distribution of genetic variation, we focused on discrimination of the 14 most frequent HLA-A, 23 HLA-B, and 12 HLA-C alleles that provide population coverage ranging from 80 to 90% in East African populations (see insets in Figure 1). None of the minor alleles was represented at allelic frequencies larger than 0.03, and even though they might have an impact at the individual level they are unlikely to have a significant influence at the population level [45]; due to statistical power constraints these minor alleles are of only marginal interest in molecular epidemiological studies. Furthermore, by limiting the scope of the assay to the major alleles in these populations, we could attempt to achieve a genotyping platform with a higher throughput, higher specificity, and higher sensitivity.

### Assay layout

**Figure 2:**
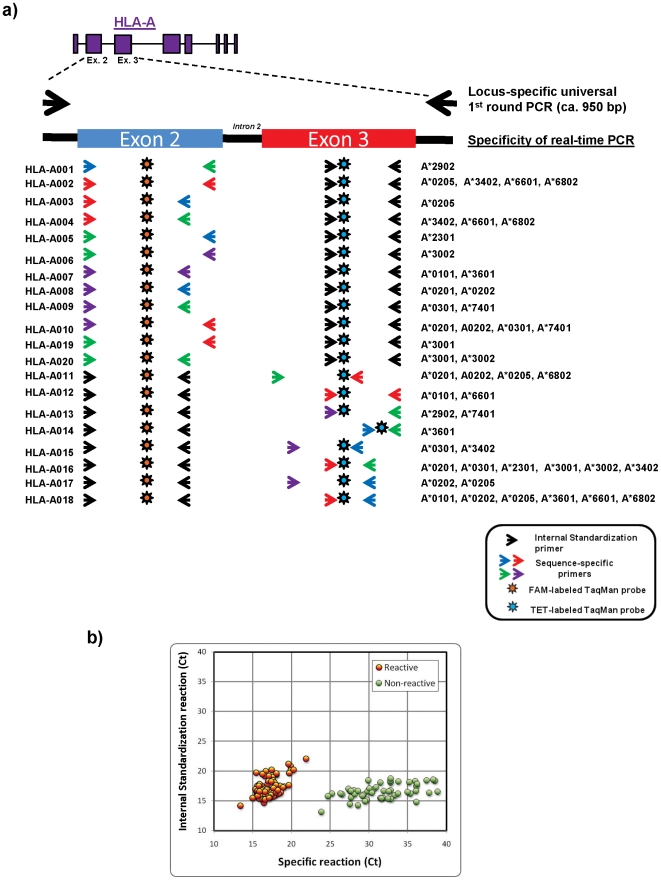
Novel sequence-specific primer (SSP) real-time PCR-based genotyping assay for HLA-A, -B, and -C in East African populations. The layout of the assay is here exemplified for the HLA-A locus, but proceeds similarly for the HLA-B and -C loci. a) After PCR amplification of a genomic region encompassing exons 2 through 3 of HLA-A using locus-specific primers, the amplicon was distributed in 20 separate multiplex SSP real-time PCRs containing sequence-specific primers (colored arrows), variation-insensitive primers (black arrows), and universal fluorescent TaqMan probes (colored stars). Sequence-specific and variation-insensitive primers targeted areas of converse exons. Sequence-specific primers were designed to more efficiently amplify defined targeted alleles (noted next to each reaction). Variation-insensitive primers were used to allow for internal standardization. b) The cross threshold (Ct) values obtained by monitoring amplification with the sequence-specific and internal standardization reagents were then used to assign samples positive or negative reactivities in each reaction (exemplified in the inset by results from reaction HLA-A018). The aggregate reactivity patterns rendered by the array of reactions were used to define the presence or absence of the addressed alleles. See text for details.

The layout of the assay is summarized in Figure 2. Genomic areas spanning exon 2 through exon 3 of the class I HLA-A, -B, and -C were respectively amplified in three separate first round PCRs, one per locus, using locus-specific primers [46], [47]. This initial amplification step prevented the subsequent interference from paralogous loci (Figure S1). These amplicons were diluted and used as templates in subsequent real-time PCR-SSPs. Each real time PCR-SSP consisted of a pair of forward and reverse sequence-specific primers, whose amplification was monitored by a fluorescent TaqMan probe targeting a conserved region encompassed by the primers. For internal standardization, a parallel real-time PCR targeted an invariant region in the converse exon within the same first-round amplicon template. The difference in amplification efficiency between the sequence-specific and the internal standardization reactions, measured as the respective Ct values, was used to assign a positive or negative reactivity to each reaction. In total, 31, 50, and 26 different primers and 7 probes (Table 1, Table 2 and Table 3) were utilized in 20 HLA-A, 46 HLA-B, and 15 HLA-C typing reactions, respectively (Table 4, Table 5 and Table 6). While some of the reactions were specific for several alleles (e.g., reactions 016 and 018 in the HLA-A locus), other reactions exhibited reactivity with only few (e.g., reactions 008 and 009 in the HLA-A locus) or a single allele (e.g., reactions 001 and 003 in the HLA-A locus) (Table 7, Table 8 and Table S1).

**Table 1:** Primers and probes used in the Class I HLA-A typing platform.

Oligonucleotides		Sequence (5′→3′)^a^	Location^b^
**Primers**			
	A226F01	GGCTCYCACTCCATGAGGTATTTC	203–226
	A228F01	GGCTCYCACTCCATGAGGTATTTCAC	203–228
	A228F02	GGCTCYCACTCCATGAGGTATTTCTA	203–228
	A228F03	GGCTCYCACTCCATGAGGTATTTCTC	203–228
	A228F04	GGCTCYCACTCCATGAGGTATTTCT[C]	203–228
	A228F05	GGCTCYCACTCCATGAGGTATTTCTT	203–228
	A228F06	GGCTCYCACTCCATGAGGTATTTCT[T]	203–228
	A400R01	TCTGTGASTGGGCCTTCACT	400–419
	A400R02	TCTGTGASTGGGCCTTCAT[A]	400–419
	A400R03	TCTGTGASTGGGCCTTCACA	400–419
	A400R04	TCTGTGASTGGGCCTTCAC[A]	400–419
	A402R01	TCTGTGASTGGGCCTTCA	402–419
	A429R01	GCAGGGTCCCCAGGTTCT	429–446
	A429R02	CGCGATCCGCAGGTTCT	429–444
	A429R03	CAGGGTCCCCAGGTTCG	429–445
	A429R04	CAGGGTCCCCAGGTCCA	429–445
	A734F01	NGTTCTCACACCVTCCAGAGG	714–734
	A756F01	GCTGCGACGTGGGGT	742–756
	A818F01	GGCAAGGATTACATCGCC	801–818
	A819F01	GGCAAGGATTACATCGCCC	801–819
	A819F02	GGCAAGGATTACATCGCC[C]	801–819
	A819F03	GGCAAGGATTACATCGCC[T]	801–819
	A898R01	RCTCTCMRCTGCTCCGCC[T]	898–916
	A898R02	RCTCTCMRCTGCTCCGCCA	898–916
	A910F01	TCCATTCGGCGGAGCAACG	892–910
	A910R02	GCCCTCCAGGTAGRCTCTC[C]	910–929
	A910R03	GCCCTCCAGGTAGGCTCTC[A]	910–929
	A930R01	GAGCCMSTCCACGCACGT	930–947
	A930R02	GAGCCMSTCCACGCACG[T]	930–947
	A930R03	AGCCMSTCCACGCACC[G]	930–946
	A932R01	GAGCCMSTCCACGCAC	932–947
**Probes**			
	A278P01	FAM-CGCACAAACTGCATGTCGTCCACGTAGCC-BHQ1	278–306
	A846P01	TET-TCTGAGCCGCCATGTCCGCCGC-BHQ1	846–867
	A921P01	TET-CTGGAGGGCTGCTGCGTGGAGTGG-BHQ1	921–944

a Locked-nucleic acid (LNA) bases are depicted between brackets.

b Position relative to reference sequence NC_000006.10 30018310–30021633 (www.ncbi.nlm.nih.gov).

**Table 2:** Primers and probes used in the Class I HLA-B typing platform.

Oligonucleotides		Sequence (5′→3′)^a^	Location^b^
**Primers**	B224F01	GCTCCCACTCCATGAGGTATTTC	202–224
	B225F01	GCTCCCACTCCATGAGGTATTTC[C]	202–225
	B225F02	GCTCCCACTCCATGAGGTATTTC[G]	202–225
	B225F03	GCTCCCACTCCATGAGGTATTTC[T]	202–225
	B231F01	CCCCACTCCATGAAGTATTTCNACACC[G]	204–231
	B234F01	CCACTCCATGAAGTATTTCNACACCNCC[A]	206–234
	B270F01	GGAGCCCCGCTTCATC[A]	254–270
	B270F02	GGAGCCCCGCTTCATC[G]	254–270
	B270F03	GGAGCCCCGCTTCATCT	254–270
	B270F04	GGAGCCCCGCTTCATC[T]	254–270
	B350R01	GCCCCTCCTGCTCTATCCAT	350–369
	B350R02	GCCCCTCCTGCTCTATCCA[C]	350–369
	B408R01	GGCTCTCTCGGTMAGTCTGTGTGT[T]	408–432
	B429F01	TCTCTCGGTMAGTCTGTGCCT[G]	408–429
	B429F02	TCTCTCGGTMAGTCTGTGCGGA	408–429
	B445R01	CGCTCTGGTTGTAGTAGCCGC	445–465
	B445R02	CGCTCTGGTTGTAGTAGCCG[C]	445–465
	B445R03	CCTCYCTCTGGTTGTAGTAGCCSGM	445–445
	B445R04	CCTCTCTCTGGTTGTAGTAGCGGA	445–468
	B734F01	CAGGGTCTCACATCATCCAG[A]	714–734
	B734F02	GGGTCTCACATCATCCAG[T]	716–734
	B734F03	CAGGGTCTCACATCATCCAG[T]	714–734
	B751F01	CAGANSATGTAYGGCTGCGAC	731–751
	B752F01	CAGANSATGTAYGGCTGCGACC	731–752
	B752F02	CAGANSATGTAYGGCTGCGAC[C]	731–752
	B752F03	CAGANSATGTAYGGCTGCGAC[G]	731–752
	B785F01	CCTCCTCCACGGGCAT[A]	769–785
	B785F02	CCTCCTCCACGGGCAT[G]	769–785
	B792F01	CGCGGGCATAACCAGT[A]	776–792
	B792F02	CGGGCATGACCAGT[C]	778–792
	B792F03	GCGGGCATGACCAGT[C]	777–792
	B793F01	CGGGCATGACCAGGA[C]	778–793
	B793F02	CGCGGGCATGACCAGTA[C]	776–793
	B793F03	GCGGGCATGACCAGTTA	777–793
	B793F04	GCGGGCATGACCAGTT[A]	777–793
	B793F05	CGCGGGTATAACCAGTT[C]	776–793
	B859R01	GGTGATCTGAGCCGCG	859–874
	B859R02	GGTGATCTGAGCCGCC	859–874
	B859R03	GGTGATCTGAGCCGC[C]	859–874
	B860R01	CTGGGTGATCTGAGCCGC	860–877
	B879R01	GCCGCCTCCMACTTGC	879–894
	B879R02	GCCGCCTCCMACTTG[C]	879–894
	B879R03	GGCCGCCTCCMACTTGA	879–895
	B911R01	CCTCCAGGTAGGTAGGCTCTCCG	911–933
	B911R02	CCTCCAGGTAGGTAGGCTCTCAG	911–933
	B911R03	CCTCCAGGTAGGTAGGCTCTCA[G]	911–933
	B911R04	CCTCCAGGTAGGTAGGCTCTGTC	911–933
	B932R01	GAGCAACTCCACGCACGT	932–949
	B932R02	GAGCAACTCCACGCACAG	932–949
	B932R03	GAGCAACTCCACGCACA[G]	932–949
**Probes**	B320P01	FAM-CWGTACGTGAGGTTCGACAGCGACGCC -BHQ1	294–320
	B805P01	TET-CAAGGATTACATCGCCCTGAACGAGGACCTG-BHQ1	805–835

a Locked-nucleic acid (LNA) bases are depicted between brackets.

b Position relative to reference sequence NC_000006.10 C31432914–31429628 (www.ncbi.nlm.nih.gov).

**Table 3:** Primers and probes used in the Class I HLA-C typing platform.

Oligonucleotides		Sequence (5′→3′)^a^	Location^b^
**Primers**			
	C235F01	CTCCCACTCCATGAGGTATTTCG[A]	212–235
	C242F01	CCATGAGGTATTTCTYCACATCC	220–242
	C255F01	TGTGTCCCGGCCCA	242–255
	C255F02	YGTGTCCCGGCCCG	242–255
	C329F01	GTTCGACAGCGACGCC	314–329
	C426R01	GCAGGTTCACTCGGTCAGT	426–444
	C427R01	CGCAGGYTCACTCGGTCAG	427–445
	C439R01	GCAGTTTCCGCAGGT	439–453
	C439R02	GCAGGTTCCGCAGG[C]	439–453
	C755F01	TCACCCTCCAGAGGATGTA	737–755
	C755F02	TCACCCTCCAGAGGATGTC	737–755
	C798F01	CCTCCTCCGCGGGTAT	783–798
	C799F01	CTCCTCCGCGGGTATG	784–799
	C806F01	CCGCGGGTATGACCAGT[A]	789–806
	C806F02	CCGCGGGTATGACCAGTC	789–806
	C806F03	CCGCGGGTATGACCAGT[C]	789–806
	C806F04	CCGCGGGTATAACCAGTT	789–806
	C925R01	CTCCAGGTAGGCTCTCTG	925–942
	C925R02	CTCCAGGTAGGCTCTCCG	925–942
	C925R03	CTCCAGGTAGGCTCTCAG	925–942
	C925R05	CTCCAGGTAGGCTCTGTC	925–942
	C925R06	CTCCAGGTAGGCTCTCCA	925–942
	C946R01	AGCCACTCCACGCACG[T]	946–962
	C946R02	AGCCACTCCACGCACAG	946–962
	C946R03	AGCCACTCCACGCACT[C]	946–962
	C948R01	GAGCCACTCCACGCAC	948–963
**Probes**			
	C380P01	FAM-CGCTTGTACTTCTGTGTCTCCCGGTCCCAATACTCC-BHQ1	380–415
	C818P01	TET-AGRTCCTCGTTCAGGGCGATGTAATCCTTGC-BHQ1	818–848

a Locked-nucleic acid (LNA) bases are depicted between brackets.

b Position relative to reference sequence NC_000006.10 31347834–31344508 (www.ncbi.nlm.nih.gov).

**Table 4:** Primer and probe combinations Class HLA-A typing reactions by real-time PCR.

Master Mix	Specific reaction			Internal standard reaction			Specificity^b^
	Primers^a^		Probe	Primers		Probe	
	Forward	Reverse		Forward	Reverse		
**HLA-A 001**	A228F01	A429R03	A278P01	A818F01	A932R01	A846P01	A*2902
**HLA-A 002**	A228F02	A429R04	A278P01	A818F01	A932R01	A846P01	A*0205, A*3402, A*6601, A*6802
**HLA-A 003**	A228F02	A400R01	A278P01	A818F01	A932R01	A846P01	A*0205
**HLA-A 004**	A228F02	A400R03	A278P01	A818F01	A932R01	A846P01	A*3402, A*6601, A*6802
**HLA-A 005**	A228F04	A429R02	A278P01	A818F01	A932R01	A846P01	A*2301
**HLA-A 006**	A228F03	A429R01	A278P01	A818F01	A932R01	A846P01	A*3002
**HLA-A 007**	A228F05	A400R02	A278P01	A818F01	A932R01	A846P01	A*0101, A*3601
**HLA-A 008**	A228F06	A400R01	A278P01	A818F01	A932R01	A846P01	A*0201, A*0202
**HLA-A 009**	A228F06	A400R04	A278P01	A818F01	A932R01	A846P01	A*0301, A*7401
**HLA-A 010**	A228F06	A429R04	A278P01	A818F01	A932R01	A846P01	A*0201, A*0202, A*0301, A*7401
**HLA-A 011**	A734F01	A898R02	A846P01	A226F01	A402R01	A278P01	A*0201, A*0202, A*0205, A*6802
**HLA-A 012**	A819F02	A930R03	A846P01	A226F01	A402R01	A278P01	A*0101, A*6601
**HLA-A 013**	A819F03	A930R01	A846P01	A226F01	A402R01	A278P01	A*2902, A*7401
**HLA-A 014**	A910F01	A930R02	A921P01	A226F01	A402R01	A278P01	A*3601
**HLA-A 015**	A756F01	A898R01	A846P01	A226F01	A402R01	A278P01	A*0301, A*3402
**HLA-A 016**	A819F02	A910R03	A846P01	A226F01	A402R01	A278P01	A*0201, A*0301, A*2301, A*3001, A*3002, A*3402
**HLA-A 017**	A756F01	A910R02	A846P01	A226F01	A402R01	A278P01	A*0202, A*0205
**HLA-A 018**	A819F01	A910R02	A846P01	A226F01	A402R01	A278P01	A*0101, A*0202, A*0205, A*3601, A*6601, A*6802
**HLA-A 019**	A228F04	A429R04	A278P01	A818F01	A932R01	A846P01	A*3001
**HLA-A 020**	A228F04	A400R04	A278P01	A818F01	A932R01	A846P01	A*3001, A*3002

a primer and probes names refer to Table 1.

b Only addressed alleles are indicated.

**Table 5:** Primer and probe combinations Class HLA-B typing reactions by real-time PCR.

Master Mix	Specific reaction			Internal standard reaction			Specificity^b^
	Primers^a^		Probe	Primers		Probe	
	Forward	Reverse		Forward	Reverse		
**HLA-B 001**	B225F01	B350R01	B320P01	B751F01	B860R01	B805P01	B*4101, B*4501, B*4901
**HLA-B 002**	B225F01	B408R01	B320P01	B751F01	B860R01	B805P01	B*1801, B*4101, B*4501, B*4901
**HLA-B 003**	B225F02	B350R02	B320P01	B751F01	B860R01	B805P01	B*0801
**HLA-B 004**	B225F03	B429F01	B320P01	B751F01	B860R01	B805P01	B*0702, B*4201, B*8101
**HLA-B 005**	B231F01	B429F02	B320P01	B751F01	B860R01	B805P01	B*1516, B*5701, B*5703, B*5801, B*5802
**HLA-B 006**	B231F01	B445R02	B320P01	B751F01	B860R01	B805P01	B*0801, B*1402, B*1503, B*1510, B*3501, B*4101, B*4501
**HLA-B 007**	B231F01	B445R04	B320P01	B751F01	B860R01	B805P01	B*1302, B*1516, B*4403, B*4901, B*5101 B*5301, B*5701, B*5703, B*5801, B*5802
**HLA-B 008**	B234F01	B445R02	B320P01	B751F01	B860R01	B805P01	B*0801, B*1503, B*1510, B*3501, B*4101, B*4501
**HLA-B 009**	B270F01	B408R01	B320P01	B751F01	B860R01	B805P01	B*1302, B*4101, B*4403, B*4501, B*4901
**HLA-B 010**	B270F01	B445R01	B320P01	B751F01	B860R01	B805P01	B*4101, B*4501
**HLA-B 011**	B270F01	B445R04	B320P01	B751F01	B860R01	B805P01	B*1302, B*2703, B*4403, B*4901
**HLA-B 012**	B270F02	B350R01	B320P01	B751F01	B860R01	B805P01	B*1516, B*3501, B*5301, B*5701, B*5703, B*5801, B*5802
**HLA-B 013**	B270F02	B408R01	B320P01	B751F01	B860R01	B805P01	B*3501, B*5301
**HLA-B 014**	B270F02	B445R02	B320P01	B751F01	B860R01	B805P01	B*3501
**HLA-B 015**	B270F03	B429F01	B320P01	B751F01	B860R01	B805P01	B*0702, B*4201, B*4202, B*8101
**HLA-B 016**	B270F03	B445R02	B320P01	B751F01	B860R01	B805P01	B*0702, B*0801, B*1402, B*1503, B*1510, B*1801, B*4201, B*4202, B*8101
**HLA-B 017**	B270F04	B445R04	B320P01	B751F01	B860R01	B805P01	B*5101
**HLA-B 018**	B734F01	B859R01	B805P01	B224F01	B445R03	B320P01	B*0801, B*1801, B*3501, B*4201, B*4202, B*4403, B*5301, B*5801
**HLA-B 019**	B734F02	B932R02	B805P01	B224F01	B445R03	B320P01	B*5802
**HLA-B 020**	B734F03	B911R02	B805P01	B224F01	B445R03	B320P01	B*1402, B*5802
**HLA-B 021**	B752F01	B879R02	B805P01	B224F01	B445R03	B320P01	B*1516, B*3501, B*4501, B*4901, B*5301, B*5801, B*5802
**HLA-B 022**	B752F02	B859R01	B805P01	B224F01	B445R03	B320P01	B*1302, B*3501, B*4501, B*4901, B*5301, B*5801, B*5802
**HLA-B 023**	B752F02	B911R04	B805P01	B224F01	B445R03	B320P01	B*4501
**HLA-B 024**	B752F03	B859R02	B805P01	B224F01	B445R03	B320P01	B*0702, B*1503, B*1510, B*2703, B*5701, B*5703, B*8101
**HLA-B 025**	B752F03	B879R01	B805P01	B224F01	B445R03	B320P01	B*0702, B*0801, B*1402, B*1503, B*1510, B*1801, B*2703, B*4101, B*4201, B*4202, B*4403, B*5101, B*5701, B*5703, B*8101
**HLA-B 026**	B752F03	B932R03	B805P01	B224F01	B445R03	B320P01	B*1503, B*1510, B*4403, B*5101, B*5701, B*5703
**HLA-B 027**	B785F01	B879R01	B805P01	B224F01	B445R03	B320P01	B*0801, B*4101, B*4201, B*4202, B*5101, B*5703, B*8101
**HLA-B 028**	B785F01	B911R02	B805P01	B224F01	B445R03	B320P01	B*1302, B*5101, B*5703, B*8101
**HLA-B 029**	B785F01	B932R03	B805P01	B224F01	B445R03	B320P01	B*5101, B*5703
**HLA-B 030**	B785F02	B879R01	B805P01	B224F01	B445R03	B320P01	B*0702, B*1503, B*1510, B*1516, B*1801, B*3501, B*5301, B*5701, B*5801, B*5802
**HLA-B 031**	B785F02	B911R02	B805P01	B224F01	B445R03	B320P01	B*1503, B*1510, B*1516, B*1801, B*3501, B*5301, B*5701, B*5801, B*5802
**HLA-B 032**	B785F02	B932R03	B805P01	B224F01	B445R03	B320P01	B*1503, B*1510, B*1516, B*3501, B*5301, B*5701, B*5801, B*5802
**HLA-B 033**	B793F01	B859R01	B805P01	B224F01	B445R03	B320P01	B*4403
**HLA-B 034**	B793F01	B879R02	B805P01	B224F01	B445R03	B320P01	B*2703, B*4403
**HLA-B 035**	B793F01	B911R03	B805P01	B224F01	B445R03	B320P01	B*2703, B*4403
**HLA-B 036**	B792F01	B911R02	B805P01	B224F01	B445R03	B320P01	B*1510, B*5101, B*5703, B*8101
**HLA-B 037**	B792F01	B911R04	B805P01	B224F01	B445R03	B320P01	B*0801, B*4101, B*4201, B*4202
**HLA-B 038**	B792F01	B932R02	B805P01	B224F01	B445R03	B320P01	B*1510, B*5101, B*5703
**HLA-B 039**	B793F02	B911R01	B805P01	B224F01	B445R03	B320P01	B*0702
**HLA-B 040**	B792F02	B911R02	B805P01	B224F01	B445R03	B320P01	B*1503, B*1516, B*1801, B*3501, B*5301, B*5701, B*5801, B*5802
**HLA-B 041**	B792F02	B932R02	B805P01	B224F01	B445R03	B320P01	B*1503, B*1516, B*3501, B*5301, B*5701, B*5801, B*5802
**HLA-B 042**	B792F03	B859R02	B805P01	B224F01	B445R03	B320P01	B*1503, B*1516, B*5701
**HLA-B 043**	B793F03	B879R03	B805P01	B224F01	B445R03	B320P01	B*1302
**HLA-B 044**	B793F04	B911R02	B805P01	B224F01	B445R03	B320P01	B*1302, B*4901
**HLA-B 045**	B793F05	B932R01	B805P01	B224F01	B445R03	B320P01	B*1402
**HLA-B 046**	B752F02	B859R03	B805P01	B224F01	B445R03	B320P01	B*1516

a primer and probes names refer to Table 2.

b Only addressed alleles are indicated.

**Table 6:** Primer and probe combinations Class HLA-C typing reactions by real-time PCR.

Master Mix	Specific reaction			Internal standard reaction			Specificity^b^
	Primers^a^		Probe	Primers		Probe	
	Forward	Reverse		Forward	Reverse		
**HLA-C 001**	C235F01	C439R01	C380P01	C798F01	C948R01	C818P01	Cw*0602, Cw*1801
**HLA-C 002**	C235F01	C439R02	C380P01	C798F01	C948R01	C818P01	Cw*0701, Cw*0702, Cw*0704
**HLA-C 003**	C242F01	C439R01	C380P01	C798F01	C948R01	C818P01	Cw*0401
**HLA-C 004**	C255F02	C439R02	C380P01	C798F01	C948R01	C818P01	Cw*0302, Cw*0304, Cw*0701, Cw*0702, Cw*0704, Cw*0802, Cw*1601
**HLA-C 005**	C806F02	C925R06	C818P01	C329F01	C427R01	C380P01	Cw*0210, Cw*0602
**HLA-C 006**	C806F03	C946R01	C818P01	C329F01	C427R01	C380P01	Cw*0602, Cw*0701, Cw*0702, Cw*1601
**HLA-C 007**	C806F04	C946R03	C818P01	C329F01	C427R01	C380P01	Cw*1701
**HLA-C 008**	C806F01	C946R02	C818P01	C329F01	C427R01	C380P01	Cw*0304
**HLA-C 009**	C799F01	C925R05	C818P01	C329F01	C427R01	C380P01	Cw*0704
**HLA-C 010**	C755F02	C925R03	C818P01	C329F01	C427R01	C380P01	Cw*0702
**HLA-C 011**	C799F01	C925R01	C818P01	C329F01	C427R01	C380P01	Cw*1601
**HLA-C 012**	C806F04	C925R02	C818P01	C329F01	C427R01	C380P01	Cw*0401, Cw*0802, Cw*1801
**HLA-C 013**	C255F01	C426R01	C380P01	C798F01	C948R01	C818P01	Cw*0210
**HLA-C 014**	C806F03	C946R02	C818P01	C329F01	C427R01	C380P01	Cw*0302
**HLA-C 015**	C755F01	C925R03	C818P01	C329F01	C427R01	C380P01	Cw*0302, Cw*0304, Cw*0701, Cw*1701

a primer and probes names refer to Table 3.

b Only addressed alleles are indicated.

**Table 7:** Reactivity patterns for HLA-A addressed alleles.

Reaction	HLA-A Allele													
	A*0101	A*0201	A*0202	A*0205	A*0301	A*2301	A*2902	A*3001	A*3002	A*3402	A*3601	A*6601	A*6802	A*7401
**HLA-A 001**							X							
**HLA-A 002**				X						X		X	X	
**HLA-A 003**				X										
**HLA-A 004**										X		X	X	
**HLA-A 005**						X								
**HLA-A 006**									X					
**HLA-A 007**	X										X			
**HLA-A 008**		X	X											
**HLA-A 009**					X									X
**HLA-A 010**		X	X		X									X
**HLA-A 011**		X	X	X									X	
**HLA-A 012**	X											X		
**HLA-A 013**							X							X
**HLA-A 014**											X			
**HLA-A 015**					X					X				
**HLA-A 016**		X			X	X		X	X	X				
**HLA-A 017**			X	X										
**HLA-A 018**	X		X	X							X	X	X	
**HLA-A 019**								X						
**HLA-A 020**								X	X					

**Table 8:** Reactivity patterns for HLA-C addressed alleles.

Reaction	HLA-C Allele											
	Cw*0210	Cw*0302	Cw*0304	Cw*0401	Cw*0602	Cw*0701	Cw*0702	Cw*0704	Cw*0802	Cw*1601	Cw*1701	Cw*1801
**HLA-C 001**					**X**							**X**
**HLA-C 002**						**X**	**X**	**X**				
**HLA-C 003**				**X**								
**HLA-C 004**		**X**	**X**			**X**	**X**	**X**	**X**	**X**		
**HLA-C 005**	**X**				**X**							
**HLA-C 006**					**X**	**X**	**X**			**X**		
**HLA-C 007**											**X**	
**HLA-C 008**			**X**									
**HLA-C 009**								**X**				
**HLA-C 010**							**X**					
**HLA-C 011**										**X**		
**HLA-C 012**				**X**					**X**			**X**
**HLA-C 013**	**X**											
**HLA-C 014**		**X**										
**HLA-C 015**		**X**	**X**			**X**					**X**	

The arrays of reactions were designed so that each of the 105 HLA-A and 78 HLA-C individual genotypes comprising the addressed alleles had a unique aggregate reactivity pattern (Table S2, Table S3, and Table S4). In the case of HLA-B, due to the allele complexity of the locus, 273/276 distinct patterns were attained, as the following pairs of addressed genotypes shared common reactivity patterns: B*4201/B*4202 and B*4201/B*4201; B*0702/B*4202 and B*0702/B*4201; and B*4201/B*8101 and B*4202/B*8101. Note that, while alleles B*4201 and B*4202 exhibit an extremely high degree of sequence identity, (i.e., differing only by a single non-synonymous change at nucleotide position 225: TAC and CAC, respectively) [48], it was possible to discriminate between carriers and non-carriers of either of these two alleles in the setting of all 39 other genotypes that involved addressed alleles.

### Assay Validation

The assay is intended for use in discriminating carriers of the most common HLA alleles in East Africa from non-carriers. A panel of 175 specimens sampled in Kampala, Uganda, previously characterized by SBT [33], was used to assess the performance of the platform (see Table S5 for a complete list of the genotypes). Performance of the HLA-A typing system was tested on 125 samples representing 63 different genotypes, composed exclusively of addressed alleles (Table 9). Carriers and non-carriers of all 14 addressed alleles could be unequivocally discriminated, rendering genotypes that were fully concordant with those obtained by SBT. Similarly, the 141 samples whose genotypes were composed exclusively of addressed HLA-B alleles were typed with 100% sensitivity and specificity. Note that in this case the validation panel represented 83 different genotypes, combining 21/23 addressed alleles. HLA-B*2703 and B*5701 were not represented in the current panel, but the assay was able to correctly detect them in specimens from Tanzania and Kenya that had been identified as carriers of these alleles by SBT (data not shown). Finally, 151 specimens that were exclusively carriers of the 12 addressed HLA-C alleles and that represented 59 different genotypes, were typed with this novel platform. Obtained results were fully concordant with those of SBT.

**Table 9:** Performance of typing platform on validation panel.

Class I HLA Allele	Individuals bearing the allele(n)^a^	Performance			
		Sensitivity	Specificity	Positive predictive value	Negative predictive value
A*0101	19	100.0%	100.0%	100.0%	100.0%
A*0201	35	100.0%	100.0%	100.0%	100.0%
A*0202	13	100.0%	100.0%	100.0%	100.0%
A*0205	4	100.0%	100.0%	100.0%	100.0%
A*0301	13	100.0%	100.0%	100.0%	100.0%
A*2301	23	100.0%	100.0%	100.0%	100.0%
A*2902	13	100.0%	100.0%	100.0%	100.0%
A*3001	17	100.0%	100.0%	100.0%	100.0%
A*3002	27	100.0%	100.0%	100.0%	100.0%
A*3402	4	100.0%	100.0%	100.0%	100.0%
A*3601	9	100.0%	100.0%	100.0%	100.0%
A*6601	18	100.0%	100.0%	100.0%	100.0%
A*6802	17	100.0%	100.0%	100.0%	100.0%
A*7401	31	100.0%	100.0%	100.0%	100.0%
B*0702	13	100.0%	100.0%	100.0%	100.0%
B*0801	12	100.0%	100.0%	100.0%	100.0%
B*1302	4	100.0%	100.0%	100.0%	100.0%
B*1402	10	100.0%	100.0%	100.0%	100.0%
B*1503	25	100.0%	100.0%	100.0%	100.0%
B*1510	15	100.0%	100.0%	100.0%	100.0%
B*1516	1	100.0%	100.0%	100.0%	100.0%
B*1801	5	100.0%	100.0%	100.0%	100.0%
B*3501	4	100.0%	100.0%	100.0%	100.0%
B*4101	3	100.0%	100.0%	100.0%	100.0%
B*4201	21	100.0%	100.0%	100.0%	100.0%
B*4202	1	100.0%	100.0%	100.0%	100.0%
B*4403	1	100.0%	100.0%	100.0%	100.0%
B*4501	32	100.0%	100.0%	100.0%	100.0%
B*4901	14	100.0%	100.0%	100.0%	100.0%
B*5101	3	100.0%	100.0%	100.0%	100.0%
B*5301	34	100.0%	100.0%	100.0%	100.0%
B*5703	9	100.0%	100.0%	100.0%	100.0%
B*5801	19	100.0%	100.0%	100.0%	100.0%
B*5802	29	100.0%	100.0%	100.0%	100.0%
B*8101	14	100.0%	100.0%	100.0%	100.0%
Cw*0210	26	100.0%	100.0%	100.0%	100.0%
Cw*0302	9	100.0%	100.0%	100.0%	100.0%
Cw*0304	21	100.0%	100.0%	100.0%	100.0%
Cw*0401	47	100.0%	100.0%	100.0%	100.0%
Cw*0602	55	100.0%	100.0%	100.0%	100.0%
Cw*0701	46	100.0%	100.0%	100.0%	100.0%
Cw*0702	11	100.0%	100.0%	100.0%	100.0%
Cw*0704	6	100.0%	100.0%	100.0%	100.0%
Cw*0802	13	100.0%	100.0%	100.0%	100.0%
Cw*1601	15	100.0%	100.0%	100.0%	100.0%
Cw*1701	23	100.0%	100.0%	100.0%	100.0%
Cw*1801	10	100.0%	100.0%	100.0%	100.0%

a Only samples from individuals bearing fully-addressed genotypes at the corresponding locus are reported (HLA-A: n = 125; HLA-B: n = 141; HLA-C: n = 151).

Within the validation panel, some of the specimens contained at least one allele not addressed in the current platform (Table S5). These genotypes were represented by 50, 34, and 24 samples for the HLA-A, -B, and -C loci, respectively. We proceeded to assess how the assay would perform on these samples. The obtained results varied depending on the nature of the non-addressed alleles, and can be grouped into four main categories (Table S6). First, there were the non- addressed alleles that fully shared a reactivity pattern with addressed alleles. In this category we could mention HLA-A*0103, HLA-A*2901, HLA-A*3009, HLA-A*7403, HLA-B*1803, HLA-B*1537, HLA-Cw*0407 and HLA-Cw*0622, which, respectively, reacted exactly like the addressed alleles HLA-A*0101, HLA-A*2902, HLA-A*3002, HLA-A*7401, HLA-B*1801, HLA-B*1510, HLA-Cw*0401 and HLA-Cw*0602. These non- addressed alleles will always be typed by the platform as their cognate addressed alleles. In a second category, we included those non-addressed alleles that had a reactivity pattern closely resembling that of one of the addressed alleles, with the addition or absence of one or two reactions. For instance, non-addressed allele HLA-A*6801 shared the reactivity with A*6802 in reactions HLA-A 002, 004, and 018 but differed from the latter in having additional reactivity at reaction 017 and the absence of reactivity at 011. In the setting of most heterozygote genotypes, these minor differences in reactivity would be eclipsed by the superimposing reactivity pattern of the accompanying allele. For the most part, these non-addressed alleles could not be distinguished from the cognate addressed alleles. Other cognate pairs of addressed and non-addressed alleles falling within this category included HLA-A*2301/HLA-A*2402, HLA-A*7401/HLA-A*3201, HLA-A*3002/HLA-A*3004, HLA-B*5703/HLA-B*5702, and HLA-Cw*0602/HLA- Cw*1203. A third category included those non-addressed alleles that reacted in only one or few reactions, rendering reactivity patterns that were eclipsed by most addressed alleles. In most of these cases, samples would be genotyped as homozygous for the identified addressed allele. Examples of this category included HLA-A*3104, HLA-B*4415, HLA-Cw*0804, and HLA-Cw*1505. Finally, some non-addressed alleles had a very distinctive reactivity pattern that allowed their detection in most settings. However, these variants were not included in the original design of the platform and therefore, there might be some relevant genotypic settings in which they might not be unequivocally genotyped. The main representative of this last category was HLA-A*0214.

Three main points are noteworthy about the aforementioned non-addressed alleles. They tend to be found at extremely low frequencies in reports from East African populations [32], [33], and thus their exclusion from the original assay design. Secondly, the observed reactivity patterns were reproducible and consistent with those expected based on their sequence. Thirdly, in most instances the presence of a non-addressed allele was not an obstacle for the adequate genotyping of the accompanying addressed alleles.

Overall, the novel genotyping platform exhibited a 100% sensitivity, specificity, positive predictive value (PPV) and negative predictive value (NPV) on specimens that were exclusively carriers of the 14, 23, and 12 addressed HLA-A, -B, and -C alleles, respectively. Additionally, the assay was able to correctly discriminate carriers from non-carriers of these variants even when they were part of genotypes that contained non-addressed alleles. The performance of the assay on the complete validation panel, including carriers of at least one non-addressed allele, is shown in Table S7. The sensitivity and NPV remained at 100% for all the addressed alleles. The specificity and PPV was 100% for all but 14 alleles. For the remainder, the sensitivity was 99.3–99.9% (9 alleles), 97.6–98.6% (4 alleles) and 87.1% for HLA-Cw*0701. The PPV of these alleles was 91.4–97.0% (8 alleles), 85.7–88.0% (3 alleles), and 60.0–78.3% (3 alleles). The most common interfering factor in the latter was the presence of non-addressed alleles which differed from the cognate addressed alleles by only one nucleotide base (see notes at the foot of Table S7 for details).

### Class I HLA genetic diversity in Mbeya, Tanzania

Following the development and validation of the real-time PCR-SSP platform, we performed a field test of this assay using a set of specimens (n = 174) from Tanzania, an East African country that to date has not been subject to systematic class I HLA genetic characterization. Samples proceeded from a cohort development study that was conducted in preparation of HIV vaccine trials in the southwestern region of Mbeya. In the HLA-A,-B, and -C loci, 174/174 (100%) and 173/174 (99.4%) and 173/174 (99.4%) samples yielded interpretable reactivity patterns, respectively. The number of samples that were carriers of at least one addressed allele were 171/174 (98.3%) in the HLA-A locus, 171/174 (98.3%) in the HLA-B locus, and 173/174 (99.4%) in the HLA-C locus. Observed HLA-A, -B, and -C allele frequencies are shown in Table S8. Overall, the alleles addressed by the novel platform provided a population coverage of 91.7%, 81.0%, and 94.0% in the HLA-A,-B, and -C loci, respectively. Observed genotypes did not deviate significantly from those expected under Hardy-Weinberg equilibrium (Table S9). All the major allelic lineages previously reported in East Africa were represented in the studied Tanzanian sample set (Figure S2). Carrier frequencies of the majority of the addressed HLA alleles tracked very closely among Tanzanians and the other East African populations [32], [33] (e.g., A*0201, A*0301, A*3001, B*1503, B*4202, B*5701, Cw*0210). Interestingly, when compared with the other East African populations, Tanzanians exhibited the highest carriage frequency for alleles A*3002, A*3601, A*6802, B*0702, B*1510, B*5301, Cw*0401, Cw*1601, and Cw*1801. On the other hand, alleles A*0101, A*0301, A*6601, B*2703, B*5701, B*5801, Cw*0302, Cw*0602, Cw*0701 and Cw*0704 tended to be under-represented in the studied Tanzanian cohort. Finally, the location of the current Tanzanian population in the context of global class I HLA genetic diversity was explored through the calculation of pair-wise inter-population genetic distances [49], [50]. The principal component analysis (PCA) based on HLA-A, -B, and -C loci grouped the current Tanzanian population together with other reported sub-Saharan populations (Figure S3). Moreover, the dendrogram analysis evidenced the Tanzanian population as an integral part of the previously reported major East African cluster, along with the Kenyan Luo, Kenyan Nandi, and Ugandan populations [33] (Figure S4).

## Discussion

The association between genetic variation in class I HLA and the susceptibility, presentation, and outcome of infectious diseases in East Africa, and the development of preventive vaccines, are topics of high public health relevance. However, the lack of adequate tools that can provide HLA typing information with the needed level of molecular detail, in a timely and cost-effective fashion, is one of the main obstacles to conducting large epidemiological studies. This deficiency is reflected in the very low representation of East African populations in global HLA databases [51]. Here, we presented a novel platform aimed at bridging the gap between high-throughput and high-resolution genotyping. When validated against a large panel of Ugandan specimens previously typed by SBT, the new assay was able to identify sensitively and unequivocally the carriers of the addressed alleles. The novel assay was successfully implemented to investigate HLA genetic diversity in Tanzania, confirming the close relationship among populations in East Africa, and revealing population-specific aspects of the genetic diversity in the studied population.

In the current platform, we implemented the 4-digit common-allele subtype resolution. In East African populations, allelic lineages tend to be represented by two or three major variants, along with several minor ones [32], [33], and even minor sequence differences among alleles from the same family have been shown to lead to extremely opposite effects regarding cellular adaptive immune responses to infectious agents. One emblematic example calling for high-resolution genotyping is that of HLA-B*5801 and B*5802, which differ in only 3/1089 exonic nucleotide bases, and are associated *in vivo* with control of HIV replication and ineffective cellular immune responses, respectively [31]. While there are precedents for the use of real-time PCR for class I HLA genotyping [52], [53], those assays usually gave only two-digit level (i.e., allelic-group designation) typing resolution, and therefore were not adequate for immunoepidemiological studies in East African populations.

HLA loci have evolved through mutation as well as recombination [54]; thus HLA alleles cannot be defined by a single nucleotide polymorphism (SNP) but rather by an array of *cis*-linked SNPs. Breaking the *cis*-linkage among SNPs is one of the main drawbacks of some HLA typing methods (e.g., SBT) as it can hamper the typing of heterozygous individuals [43]. By basing the novel platform on the PCR-SSP method, we were able to preserve both the information about the polymorphisms and their linkage. To avoid time-consuming post-PCR detection by agarose-gel electrophoresis, which is one of the main disadvantages of conventional PCR-SSP, we opted to implement the platform using real-time PCR, where the detection of the positive reactivity is concurrent with the amplification reaction itself, in a closed system [47]. Furthermore, performing real-time PCR-SSP in a multiplex format allowed the incorporation of internal standardization, measuring in parallel the degree of sequence identity between template and primers, and the amount of template incorporated in the reaction. Due to the reaction conditions used in the real-time PCR-SSP, only amplicons shorter than 250 bp could be efficiently amplified. For this reason, only the linkage between SNPs lying in the same exon could be interrogated. Nevertheless, the information provided by these reactions was suitable for the intended use of the assay.

The high throughput, low cost, low post-PCR processing, and automation potential that characterize real-time PCR present clear advantages over other widely used techniques, such as conventional PCR-SSP or PCR-SSOP. Despite the high initial set-up cost of the infrastructure required to run real-time PCR, equipment and reagents are progressively becoming standard tools in molecular biology, especially in laboratories dedicated to genetics of infectious and autoimmune diseases, or can be found in genotyping core facilities. It is likely that the evolution of real-time PCR technologies will soon allow for implementing the current platform closer to the field, where the data is being collected [55], [56]. The interpretation of the results can be computerized by direct export of Ct values from the instrument, followed by their conversion into reactive/non-reactive binary patterns and comparison to expected reactivity patterns for addressed genotypes, and finally, the assembly into a database. The minimal need for manual data entry makes the current molecular platform ideal for epidemiologic studies. In our hands, a single operator with a fully dedicated instrument can genotype class I HLA-A, -B, and -C for ca. 50 specimens per week. Achieving a comparable throughput by conventional PCR-based techniques would require a much larger work force, dedicated to labor-intensive gel electrophoresis, interpretation, and manual data entry.

The current platform has several limitations inherent to its design, so it is not meant to replace gold-standard SBT, and it should be used only for research and not for diagnostics or therapeutics purposes. With the current assay, only addressed alleles can be detected with high sensitivity and specificity. Rare, non-addressed alleles sometimes cannot be detected, leading to overcalling of homozygotes. Assessing major deviations from Hardy-Weinberg equilibrium can help identify this problem. Alternatively, non-addressed variants may be assigned to highly related addressed alleles, and SBT may be used for further genotype confirmation.

The extreme level of genetic diversity characteristic of the HLA loci prevents the achievement of high-sensitivity and high-specificity typing of the over 2,000 class I HLA-A, -B, and -C alleles reported worldwide. However, our focusing on the 49 most common variants reported in East Africa, which provide 80–90% population coverage, offered an adequate balance between the quantity and the quality of the data that can be gathered. While many alleles found in East Africa were not addressed in the current assay, their very low representation in these populations results in their relatively low public health impact. The modular and “open source” nature of the current assay permits incorporation, by any member of the field, of further reactions that can allow for the discrimination of carriers of any given non-addressed variant deemed to be of interest. The current molecular platform was tailor-made for East Africa, and thus has an application limited only to this geographic area, which is home to more than 100 million individuals and presents high prevalence of infectious diseases including HIV/AIDS, malaria, and tuberculosis [14]. Similar platforms based on the same principles, targeting the HLA genetic diversity in other global populations (e.g., Southeast Asia), are currently being designed to support large cohort-based studies.

Using the novel typing platform, we were able to provide for the first time a detailed description of a Tanzanian population. Genetic distance analyses demonstrate that this population was highly related to other sub-Saharan groups, and more specifically, it was embedded within the previously defined major East African cluster [32], [33]. These results are concordant with recently published findings, based on the analysis of non-immunogenetic loci [57].The commonalities found between the Tanzanian, Ugandan and Kenyan populations were reflected in the presence of the same allelic lineages, defining the immunogenetic background of East African populations. On the other hand, subtle genetic differences among these groups were also evident, indicating the uniqueness of each individual population within the major cluster. Interestingly, each of these populations is home to unique genetic forms of common widely spread pathogens. For instance, HIV type-1 strains circulating in East Africa represent mostly group-M subtypes A, C, D, and a constellation of recombinant forms among them, but the genetic subtypes are differently balanced in each country [58], [59], [60], [61], [62]. Coupled with existing high-throughput viral subtyping assays [62], the current platform will be able to provide high-resolution HLA information with the needed throughput, to elucidate the underlying immunogenetic basis of this unique subtype distribution.

Among the most relevant immunoepidemiological applications for the novel genotyping platform are association studies between host genotype and disease susceptibility and outcome [31], and the analysis of host-pathogen genetic co-variation [63], [64]. Furthermore, this assay allows for the identification of large numbers of individuals who are carriers of HLA alleles of interest to support functional characterization of immune responses to pathogens [65] or vaccines [66]. High-resolution HLA typing has provided deep insight into the underlying molecular mechanisms of host-pathogen interaction [67]. East Africa is one of the world regions with the highest pathogen burdens [14], which can be mitigated by preventive vaccines. The availability of high- throughput high- resolution HLA typing platforms, such as the one presented here, will be extremely useful in the identification of correlates of immune protection and the evaluation of the effectiveness of candidate vaccines.

## Materials and Methods

### Ethics Statement

All volunteers completed informed consent, and the study was reviewed and approved by the human subject ethics and safety committees, in compliance with all relevant federal guidelines and institutional policies.

### Sequence alignment for assay development

Published class I HLA-A, -B, and -C nucleotide sequences of alleles reported in East Africa [32], [33] were retrieved from the IMGT/HLA Database (http://www.ebi.ac.uk/imgt/hla/) [68]. For each locus, alignments of nucleotide sequences representing the targeted alleles were constructed using ClustalX [69] and were manually edited using Genetic Data Environment [70]. Polymorphic sites that helped to discriminate among these alleles were identified by visual inspection. The sequence analysis was restricted to exons 2 and 3 of the HLA loci, which define the peptide-binding α1 and α2 domains, the only region for which sequences were available for all of the targeted alleles defined at the 4-digit level.

### Primer/Probe Design

Oligonucleotide primers and probes were designed using Primer Express software version 2.0 (Applied Biosystems, Foster City, CA) and PrimerSelect version 7.1.0 as implemented in the Lasergene package (DNASTAR, Madison, WI). The primers were designed so that their 3′extremes would determine their sequence specificity, their melting temperature (Tm) would be approximately 65°C to ensure uniform amplification conditions, and with minimal potential for constrained secondary structure or primer-dimer formation. TaqMan fluorescent probes, targeting highly conserved regions, were designed to serve as universal reagents that allow for kinetic read-out by real-time PCR.

### Real-time PCR-SSP

For HLA typing, 900–980 bp fragments encompassing exons 2 through 3 of HLA-A, -B, or -C were PCR amplified in three separate reactions using locus-specific primers targeting conserved regions of each respective HLA gene, as previously described [46], [47]. Briefly, the first-round PCR contained 10× PCR Gold Buffer (Applied Biosystems, Foster City, CA), 200 nM of each dNTP, 1.5 mM MgCl_2_, 400 nM of each primer (Sigma Aldrich, St. Louis, MO) [46], 1.25 U of AmpliTaq Gold DNA Polymerase (Applied Biosystems, Foster City, CA) and genomic DNA (20–100 ng) in a final volume of 50 uL. Thermocycling conditions were: 10 min at 95°C, followed by 30 cycles of 30 seconds at 95°C, 1 minute at 65°C, and 2 min at 72°C. First-round PCR products were each diluted 1000-fold in molecular-grade water for use in subsequent real-time PCR-based genotyping reactions. Corresponding first-round PCR dilutions were distributed into 20, 46 and 15 separate real-time-PCR-SSPs for the targeted variants in HLA-A, -B, and -C, respectively. Each reaction used a multiplex format designed to target both a sequence-specific region and a non-polymorphic region of the amplicon itself, for internal standardization. Amplification was monitored in real-time using TaqMan fluorescent probes. When variation was assessed in the exon 2 using polymorphism-specific primers and FAM-labeled probes, the internal standardization reaction was designed to amplify a segment of exon 3 with detection by TET-labeled probes, and vice versa for exon 3. Tables 1, Table 2, Table 3, Table 4, Table 5 and Table 6 indicate the sequences and combinations of primers and probes used for each of the typing reactions. Several primers contain locked nucleic acid (LNA) modifications at the 3′ extreme [71], [72]. Because LNAs are a class of nucleic acid analogues that have a more rigid configuration than standard oligonucleotide primers, they perform with higher specificity than standard primers, although sometimes at the expense of amplification efficiency [47]. The applicability of LNAs for each reaction was determined empirically (data not shown). Each of the genotyping real-time PCR mixtures consisted of TaqMan 2× Universal PCR Master Mix No AmpErase UNG (Applied Biosystems, Foster City, CA), 400 nM of each forward and reverse sequence-specific primers (Sigma Aldrich, St. Louis, MO and Exiqon, Vedbaek, Denmark), 400 nM of each forward and reverse universal primers (Sigma Aldrich, St. Louis, MO), 250 nM of a locus-specific probe (Sigma Aldrich, St. Louis, MO), 250 nM of an internal-standardization probe (Sigma Aldrich, St. Louis, MO), and diluted first-round PCR product, in a final volume of 6.25 uL. Samples were run in a 384-well plate format with the following thermocycling program: 10 min at 95°C followed by 60 cycles of 15 seconds at 95°C and 1 minute at 60°C. The intensity of each fluorescent probe was read automatically by the 7900HT Fast Real-time PCR System (Applied Biosystems, Foster City, CA) then analyzed and interpreted with Sequence Detection Software version 2.2.2 (Applied Biosystems, Foster City, CA) as the cycle threshold (Ct), i.e., the number of cycles required to bring the fluorescent signal generated in the reaction above a set threshold. Samples that did not cross the threshold were manually assigned a Ct of the maximum 60. In all cases, non-template controls were included where water substituted for genomic DNA. Positive reactivity for each reaction was determined by computation of the difference in Ct values between the sequence-specific and the internal-standardization reactions and comparison to empirically determined cut-offs. The calling of HLA genotypes was performed by comparing the observed aggregate reactivity patterns of real-time PCR-SSP with those deduced from the sequences of addressed alleles (Table 7, Table 8, Table S1, Table S2, Table S3, and Table S4).

### Assay validation

Assay validation was based on a panel of 175 specimens from Kampala, Uganda, previously characterized by class I HLA-A, -B, and -C SBT at the 4-digit level [33]. Genomic DNA was extracted from Epstein-Barr virus (EBV) transformed B-cell lines prepared from peripheral blood mononuclear cells (PBMCs) separated from whole blood (MagNA pure total nucleic acid extraction, Roche Diagnostics Corp., Indianapolis, IN). Detailed previous analysis had demonstrated that these specimens provided a representative sample of the HLA genetic diversity found in East Africa [33]. The samples represented 108, 114, and 80 different HLA-A, -B, and -C genotypes, respectively, and included alleles A*0101, A*0102, A*0103, A*0109, A*0123, A*0201, A*0202, A*0205, A*0214, A*0301, A*2301, A*2402, A*2601, A*2612, A*2901, A*2902, A*3001, A*3002, A*3004, A*3009, A*3101, A*3104, A*3201, A*3303, A*3402, A*3601, A*6601, A*6602, A*6603, A*6801, A*6802, A*7401, A*7403, , B*0702, B*0705, B*0801, B*1302, B*1303, B*1401, B*1402, B*1503, B*1510, B*1516, B*1531, B*1537, B*1801, B*1803, B*3501, B*3502, B*3503, B*3701, B*3910, B*3924, B*4012, B*4101, B*4102, B*4201, B*4202, B*4403, B*4415, B*4501, B*4703, B*4901, B*5001, B*5101, B*5301, B*5702, B*5703, B*5801, B*5802, B*7301, B*8101, B*8202, , Cw*0210, Cw*0302, Cw*0304, Cw*0401, Cw*0404, Cw*0407, Cw*0501, Cw*0602, Cw*0622, Cw*0701, Cw*0702, Cw*0704, Cw*0706, Cw*0718, Cw*0802, Cw*0804, Cw*1203, Cw*1402, Cw*1505, Cw*1601, Cw*1602, Cw*1701, Cw*1801. Sensitivity, specificity, PPV, and NPV of the real-time PCR-SSP platform were calculated as previously described by Altman and Bland [73], [74] using SBT as the reference method.

### Field test of the assay

A sample set from Tanzania was used to field test the real-time PCR-SSP platform. Between September 2002 to April 2003, 3096 volunteers from Mbeya (southwestern Tanzania, latitude 8°54′53″S and longitude 33°27′43″E) were enrolled in a prospective community cohort study, with the objective of assessing the suitability of different population groups for HIV vaccine cohort development. The composition of this cohort was described in detail elsewhere [58]. The study was conducted jointly by the Mbeya Regional AIDS Control Programme (Tanzanian Ministry of Health), the Department of Infectious Diseases & Tropical Medicine, Ludwigs-Maximillians University (Munich, Germany), the Walter Reed Army Institute of Research (Rockville, MD, USA), and the Henry M. Jackson Foundation for the Advancement of Military Medicine (Rockville, MD, USA). Blood samples collected from 174 randomly selected individuals, out of the 2479 participants who remained HIV sero-negative for the 42-month duration of the study, were available for HLA typing. This sample set included 110 female (63.2%), and the median age was 26 years (inter-quartile interval: 21–35 years). All of the tested participants were Black Africans residing in the Mbeya Region, and were recruited from the urban Ghana ward in Mbeya Town, and from the small rural village of Itende. Genomic DNA was extracted from peripheral blood mononuclear cells (PBMCs) separated from whole blood (MagNA pure total nucleic acid extraction, Roche Diagnostics Corp., Indianapolis, IN).

### Comparison of genetic composition among world populations

Class I HLA-A, -B, and -C allele frequencies from world populations were retrieved from the dbMHC database [75]. To facilitate the comparison between the current SBT HLA data with historical datasets, which were often described using other techniques or with other levels of molecular resolution, allele Cw*0210 was considered synonymous with Cw*0202 [76]. Similarly, for HLA-C alleles that are often not distinguishable, the previously defined allele grouping systems were applied, which include Cw*0401G, Cw*0501G, Cw*0701G, Cw*0704G, Cw*1701G, and Cw*1801G [32]. Inter-population genetic distances were estimated using the definition proposed by Cavalli-Sforza and Bodmer [49], [50], which is a measure of the level of overlap of genetic variants between pairs of populations, as implemented by the GENDIST module of PHYLIP (Phylogeny Inference Package) version 3.6 [77]. The estimated genetic distance matrixes so obtained were used to construct unrooted dendrograms through the neighbor-joining algorithm [78] as implemented in MEGA version 4 [79]. Contingency tables were tested using the Fisher's exact test and the Fisher-Freeman-Halton test using StatXact Version 6 (Cytel Software Corporation, MA). Principal component analysis (PCA) was performed using JMP®, Version 7.0.2 (SAS Institute Inc., Cary, NC) based on the calculated genetic distances.

## Supporting Information

Table S1 Reactivity patterns for HLA-B addressed alleles.
(0.03 MB XLS)

Table S2 Expected reactivity patterns for 105 HLA-A genotypes among addressed alleles.
(0.05 MB XLS)

Table S3 Expected reactivity patterns for 276 HLA-B genotypes among addressed alleles.
(0.21 MB XLS)

Table S4 Expected reactivity patterns for 78 HLA-C genotypes among addressed alleles.
(0.04 MB XLS)

Table S5 Class I HLA-A, -B, and -C genotypes of 175 samples from Kampala, Uganda, used for assay validation.
(0.04 MB XLS)

Table S6 Examples of expected and observed reactivity patterns for non-addressed genotypes. See text for details.
(0.03 MB XLS)

Table S7 Performance of typing platform on the complete sample set from Kampala, Uganda (n = 175).
(0.06 MB DOC)

Table S8 Allele frequencies for class I HLA-A,-B, and -C in Mbeya, Tanzania (2n = 348). Only alleles addressed by the current SSP-real-time PCR assay are listed. See text for details.
(0.06 MB DOC)

Table S9 Observed genotypes in Tanzania (n = 174) did not significantly differ from those expected under Hardy-Weinberg equilibrium.
(0.09 MB DOC)

Figure S1 Locus-specific pre-amplification is necessary for optimal performance of SSP-real time PCR-based HLA typing. Performance of reaction HLA-B016 (which is specific for HLA-B*0702, HLA-B*0801, HLA-B*1402, HLA-B*1503, HLA-B*1510, HLA-B*1801, HLA-B*4201, HLA-B*4202, and HLA-B*8101) on Ugandan samples a) before, and b) after subjecting to HLA-B-specific PCR pre-amplification. Red circles and black crosses depict carriers and non-carriers of the addressed polymorphisms, respectively. Non-template controls are depicted by triangles. See text for details.
(1.55 MB TIF)

Figure S2 Carrier frequencies of major class I HLA alleles in East African populations. Carrier frequencies of alleles addressed by the current platform are shown for the Kenyan Highlander [Nandi] [32] (yellow), Kenyan Lowlander [Luo] (orange) [32], Uganda [33] (green) and the current Tanzanian population (red).
(0.73 MB TIF)

Figure S3 Principal component analysis showing the location of the Mbeya, Tanzania population in the context of global class I HLA genetic diversity. Principal component analysis (PCA) based on genetic distances in HLA-A,-B, and -C loci. Reference world populations were retrieved from the dbMHC [75]. In interest of clarity, only the first and second principal components are shown, which account for 79.4% of the variance. The composition of the outlined sub-Saharan cluster is shown in detail in the inset. The expanded box shows a close up the sub-Saharan populations. Populations are labeled as follows: 1: AmericanSamoa (American Samoa, United States), 2: Amerindian (United States), 3: Ami97 (Taiwan), 4: ArabDruze (Israel), 5: Atayal (Taiwan), 6: Bari (Venezuela), 7: Brazilian Admixed (Brazil), 8: Bulgarian (Bulgaria), 9: Bunun (Taiwan), 10: Canoncito (New Mexico, United States), 11: CapeYork (Australia), 12: Chinese (China), 14: Mbeya (Tanzania), 16: Czech (Czech Republic), 17: Doggon (Mali), 18: Filipino (Phillipines), 19: Finn90 (Finland), 20: Georgian (Georgia), 21: GrooteEylandt (Australia), 22: Guarani-Kaiowa (Brazil), 23: Guarani-Nandewa (Brazil), 24: Hakka (Taiwan), 25: Irish (Ireland), 26: IsraeliJews (Irish), 27: Ivatan (Philippines), 28: JavaneseIndonesian (Singapore), 29: Kenyan142 (Kenya), 30: KenyanHighlander (Kenya), 31: KenyanLowlander (Kenya), 32: Kimberley (Australia), 33: Korean200 (South Korea), 34: Kurdish (Georgia), 35: Malay (Singapore), 36: Minnan (Taiwan), 37: NewDelhi (India), 38: African American (United States), 39: Asian American (United States), 40: Caucasian (United States), 41: Hispanic (United States), 42: Okinawan (United States), 43: Paiwan51 (Taiwan), 44: Pazeh (Taiwan), 45: Puyuma49 (Taiwan), 46: Rukai (Taiwan), 47: Kampala, Uganda (Uganda), 48: Saisiat (Taiwan), 49: Shona (Zimbabwe), 50: Siraya (Taiwan), 51: Tamil (South Africa), 52: Thai (Singapore), 53: Thao (Taiwan), 54: Toroko (Taiwan), 55: Tsou (Taiwan), 56: Tuva (Taiwan), 58: Yami (Taiwan), 59: Yuendumu (Australia), 60: Yupik (Alaska, United States), 61: Zambian (Zambia), 62: Zulu (South Africa). See text for details.
(1.26 MB TIF)

Figure S4 Dendrogram showing the location of the Mbeya, Tanzania population in the context of global class I HLA genetic diversity. The unrooted dendrogram was built using combined HLA-A,-B, -C allele frequency derived genetic distances. The expanded box shows a close up the sub-Saharan populations. Populations are labeled as follows: 1: AmericanSamoa (American Samoa, United States), 2: Amerindian (United States), 3: Ami97 (Taiwan), 4: ArabDruze (Israel), 5: Atayal (Taiwan), 6: Bari (Venezuela), 7: Brazilian Admixed (Brazil), 8: Bulgarian (Bulgaria), 9: Bunun (Taiwan), 10: Canoncito (New Mexico, United States), 11: CapeYork (Australia), 12: Chinese (China), 14: Mbeya (Tanzania), 16: Czech (Czech Republic), 17: Doggon (Mali), 18: Filipino (Phillipines), 19: Finn90 (Finland), 20: Georgian (Georgia), 21: GrooteEylandt (Australia), 22: Guarani-Kaiowa (Brazil), 23: Guarani-Nandewa (Brazil), 24: Hakka (Taiwan), 25: Irish (Ireland), 26: IsraeliJews (Irish), 27: Ivatan (Philippines), 28: JavaneseIndonesian (Singapore), 29: Kenyan142 (Kenya), 30: KenyanHighlander (Kenya), 31: KenyanLowlander (Kenya), 32: Kimberley (Australia), 33: Korean200 (South Korea), 34: Kurdish (Georgia), 35: Malay (Singapore), 36: Minnan (Taiwan), 37: NewDelhi (India), 38: African American (United States), 39: Asian American (United States), 40: Caucasian (United States), 41: Hispanic (United States), 42: Okinawan (United States), 43: Paiwan51 (Taiwan), 44: Pazeh (Taiwan), 45: Puyuma49 (Taiwan), 46: Rukai (Taiwan), 47: Kampala, Uganda (Uganda), 48: Saisiat (Taiwan), 49: Shona (Zimbabwe), 50: Siraya (Taiwan), 51: Tamil (South Africa), 52: Thai (Singapore), 53: Thao (Taiwan), 54: Toroko (Taiwan), 55: Tsou (Taiwan), 56: Tuva (Taiwan), 58: Yami (Taiwan), 59: Yuendumu (Australia), 60: Yupik (Alaska, United States), 61: Zambian (Zambia), 62: Zulu (South Africa). The Tanzanian population is depicted as a filled triangle. See text for details.
(0.86 MB TIF)
