# Supplementary material for: High-Throughput High-Resolution Class I HLA Genotyping in East Africa
Source: PLoS One. 2010 May 20;5(5):e10751. doi: 10.1371/journal.pone.0010751 (PMC2873994; doi:10.1371/journal.pone.0010751)
Supplement: Table S7 — Performance of typing platform on the complete sample set from Kampala, Uganda (n = 175). (0.06 MB DOC) [file pone.0010751.s007.doc]

Table S7. Performance of typing platform on the complete sample set from Kampala, Uganda (n=175)

| **Class I HLA Allele** | **Individuals bearing the allele (n)^a^** | **Performance^b^** | | | |
| --- | --- | --- | --- | --- | --- |
|  |  | **Sensitivity** | **Specificity** | **Positive predictive value** | **Negative predictive value** |
| A*0101 | 22 | 100.0% | 99.3% | 95.7% | 100.0% |
| A*0201 | 37 | 100.0% | 100.0% | 100.0% | 100.0% |
| A*0202 | 13 | 100.0% | 100.0% | 100.0% | 100.0% |
| A*0205 | 5 | 100.0% | 100.0% | 100.0% | 100.0% |
| A*0301 | 15 | 100.0% | 100.0% | 100.0% | 100.0% |
| A*2301 | 32 | 100.0% | 99.3% | 97.0% | 100.0% |
| A*2902 | 18 | 100.0% | 96.8% | 78.3%^c^ | 100.0% |
| A*3001 | 21 | 100.0% | 100.0% | 100.0% | 100.0% |
| A*3002 | 28 | 100.0% | 98.6% | 93.3% | 100.0% |
| A*3402 | 7 | 100.0% | 100.0% | 100.0% | 100.0% |
| A*3601 | 10 | 100.0% | 100.0% | 100.0% | 100.0% |
| A*6601 | 22 | 100.0% | 98.0% | 88.0% | 100.0% |
| A*6802 | 22 | 100.0% | 100.0% | 100.0% | 100.0% |
| A*7401 | 32 | 100.0% | 97.9% | 91.4% | 100.0% |
| B*0702 | 15 | 100.0% | 100.0% | 100.0% | 100.0% |
| B*0801 | 15 | 100.0% | 100.0% | 100.0% | 100.0% |
| B*1302 | 6 | 100.0% | 99.4% | 85.7% | 100.0% |
| B*1402 | 10 | 100.0% | 100.0% | 100.0% | 100.0% |
| B*1503 | 30 | 100.0% | 100.0% | 100.0% | 100.0% |
| B*1510 | 18 | 100.0% | 99.4% | 94.7% | 100.0% |
| B*1516 | 1 | 100.0% | 100.0% | 100.0% | 100.0% |
| B*1801 | 6 | 100.0% | 97.6% | 60.0%^d^ | 100.0% |
| B*3501 | 4 | 100.0% | 100.0% | 100.0% | 100.0% |
| B*4101 | 4 | 100.0% | 100.0% | 100.0% | 100.0% |
| B*4201 | 22 | 100.0% | 100.0% | 100.0% | 100.0% |
| B*4202 | 1 | 100.0% | 100.0% | 100.0% | 100.0% |
| B*4403 | 2 | 100.0% | 100.0% | 100.0% | 100.0% |
| B*4501 | 34 | 100.0% | 100.0% | 100.0% | 100.0% |
| B*4901 | 18 | 100.0% | 100.0% | 100.0% | 100.0% |
| B*5101 | 3 | 100.0% | 100.0% | 100.0% | 100.0% |
| B*5301 | 36 | 100.0% | 100.0% | 100.0% | 100.0% |
| B*5703 | 10 | 100.0% | 100.0% | 100.0% | 100.0% |
| B*5801 | 20 | 100.0% | 100.0% | 100.0% | 100.0% |
| B*5802 | 33 | 100.0% | 100.0% | 100.0% | 100.0% |
| B*8101 | 14 | 100.0% | 100.0% | 100.0% | 100.0% |
| Cw*0210 | 28 | 100.0% | 100.0% | 100.0% | 100.0% |
| Cw*0302 | 10 | 100.0% | 99.4% | 90.9% | 100.0% |
| Cw*0304 | 22 | 100.0% | 100.0% | 100.0% | 100.0% |
| Cw*0401 | 51 | 100.0% | 94.4% | 87.9% | 100.0% |
| Cw*0602 | 59 | 100.0% | 100.0% | 100.0% | 100.0% |
| Cw*0701 | 36 | 100.0% | 87.1% | 66.7%^e^ | 100.0% |
| Cw*0702 | 13 | 100.0% | 99.4% | 92.9% | 100.0% |
| Cw*0704 | 6 | 100.0% | 100.0% | 100.0% | 100.0% |
| Cw*0802 | 13 | 100.0% | 99.4% | 92.9% | 100.0% |
| Cw*1601 | 17 | 100.0% | 100.0% | 100.0% | 100.0% |
| Cw*1701 | 24 | 100.0% | 99.3% | 96.0% | 100.0% |
| Cw*1801 | 10 | 100.0% | 100.0% | 100.0% | 100.0% |

^a^. Tested samples include those from individuals bearing addressed and non-addressed genotypes. For the performance of the platform only on samples bearing fully-addressed genotypes, see table 4.

^b^. To calculate the performance of the SSP-real-time PCR platform, sequence-based typing was used the gold-standard method. See text for details.

^c^. Twenty-three samples were typed by real-time PCR as carriers of HLA-A*2902, 18 (78%) of which had a concordant typing by sequence-based typing (SBT). The remainder 5 samples were typed as carriers of HLA-A*2901 by SBT. A*2901 and A*2902 differ in only 1 base, in exon 2. Based on its nucleotide sequence, the expected reactivity pattern of A*2901 is identical to that of A*2902.

^d^. Ten samples were typed by real-time PCR as carriers of HLA-B*1801, 6 of which had a concordant typing by sequence-based typing (SBT). The remainder 4 samples were typed as carriers of HLA-B*1803 by SBT. B*1801 and B*1803 differ in only 1 base, in exon 2. Based on its nucleotide sequence, the expected reactivity pattern of B*1803 is identical to that of B*1801.

^e^. Fifty-four samples were typed by real-time PCR as carriers of HLA-Cw*0701, 36 (67%) of which had a concordant typing by sequence-based typing (SBT). The remainder 18 samples were typed as carriers of HLA-Cw*0718 by SBT. Cw*0701 and Cw*0718 differ in only 1 base, in exon 6, encoding the cytoplasmic domain–a region not sequenced by the vast majority of HLA-typing protocols currently in use. For this reason, the IMGT/HLA Database Exon identity and Ambiguous Typing Combinations List (release 2.28.0)assigned them Cw*07G1. Based on its nucleotide sequence, the expected reactivity pattern of Cw*0718 is identical to that of Cw*0701.
