# Supplementary material for: High-Throughput High-Resolution Class I HLA Genotyping in East Africa
Source: PLoS One. 2010 May 20;5(5):e10751. doi: 10.1371/journal.pone.0010751 (PMC2873994; doi:10.1371/journal.pone.0010751)
Supplement: Table S8 — Allele frequencies for class I HLA-A,-B, and -C in Mbeya, Tanzania (2n = 348). Only alleles addressed by the current SSP-real-time PCR assay are listed. See text for details. (0.06 MB DOC) [file pone.0010751.s008.doc]

Table S8. Allele Frequencies for class I HLA-A,-B, and -C in Mbeya, Tanzania (2n = 348). Only alleles addressed by the current SSP-real-time PCR assay are listed. See text for details

| **HLA-A** | **Allele frequency** |  | **HLA-B** | **Allele frequency** |  | **HLA-C** | **Allele frequency** |
| --- | --- | --- | --- | --- | --- | --- | --- |
| **A*0101** | 0.0287 |  | **B*0702** | 0.0690 |  | **Cw*0210** | 0.0891 |
| **A*0201** | 0.1149 |  | **B*0801** | 0.0172 |  | **Cw*0302** | 0.0029 |
| **A*0202** | 0.0402 |  | **B*1302** | 0.0115 |  | **Cw*0304** | 0.0632 |
| **A*0205** | 0.0287 |  | **B*1402** | 0.0201 |  | **Cw*0401** | 0.2241 |
| **A*0301** | 0.0230 |  | **B*1503** | 0.0891 |  | **Cw*0602** | 0.1437 |
| **A*2301** | 0.1006 |  | **B*1510** | 0.0718 |  | **Cw*0701** | 0.1178 |
| **A*2902** | 0.0460 |  | **B*1516** | 0.0029 |  | **Cw*0702** | 0.0259 |
| **A*3001** | 0.0460 |  | **B*1801** | 0.0201 |  | **Cw*0704** | 0.0172 |
| **A*3002** | 0.1207 |  | **B*2703** | 0.0000 |  | **Cw*0802** | 0.0402 |
| **A*3402** | 0.0287 |  | **B*3501** | 0.0172 |  | **Cw*1601** | 0.0718 |
| **A*3601** | 0.0862 |  | **B*4101** | 0.0057 |  | **Cw*1701** | 0.0833 |
| **A*6601** | 0.0230 |  | **B*4201** | 0.0489 |  | **Cw*1801** | 0.0603 |
| **A*6802** | 0.1351 |  | **B*4402** | 0.0057 |  |  |  |
| **A*7401** | 0.0948 |  | **B*4403** | 0.0144 |  |  |  |
|  |  |  | **B*4501** | 0.0632 |  |  |  |
|  |  |  | **B*4901** | 0.0287 |  |  |  |
|  |  |  | **B*5101** | 0.0029 |  |  |  |
|  |  |  | **B*5301** | 0.1092 |  |  |  |
|  |  |  | **B*5701** | 0.0000 |  |  |  |
|  |  |  | **B*5703** | 0.0345 |  |  |  |
|  |  |  | **B*5801** | 0.0374 |  |  |  |
|  |  |  | **B*5802** | 0.0891 |  |  |  |
|  |  |  | **B*8101** | 0.0546 |  |  |  |
