# Supplementary material for: High-Throughput High-Resolution Class I HLA Genotyping in East Africa
Source: PLoS One. 2010 May 20;5(5):e10751. doi: 10.1371/journal.pone.0010751 (PMC2873994; doi:10.1371/journal.pone.0010751)
Supplement: Table S9 — Observed genotypes in Tanzania (n = 174) did not significantly differ from those expected under Hardy-Weinberg equilibrium. (0.09 MB DOC) [file pone.0010751.s009.doc]

Table S9. Observed genotypes in Tanzania (n=174) did not significantly differ from those expected under Hardy-Weinberg equilibrium.

| **Class I HLA allele** | **Observed** | | | |  | **Expected^a^** | | | **p value^b^** |
| --- | --- | --- | --- | --- | --- | --- | --- | --- | --- |
|  | **Homozygotes** | **Heterozygotes** | **Non carriers** | **Allele frequency** |  | **Homozygotes** | **Heterozygotes** | **Non carriers** |  |
| **A*0101** | 0 | 10 | 164 | 0.0287 |  | 0 | 10 | 164 | 1.000 |
| **A*0201** | 1 | 38 | 135 | 0.1149 |  | 2 | 35 | 136 | 0.844 |
| **A*0202** | 0 | 14 | 160 | 0.0402 |  | 0 | 13 | 160 | 1.000 |
| **A*0205** | 0 | 10 | 164 | 0.0287 |  | 0 | 10 | 164 | 1.000 |
| **A*0301** | 0 | 8 | 166 | 0.0230 |  | 0 | 8 | 166 | 1.000 |
| **A*2301** | 3 | 29 | 142 | 0.1006 |  | 2 | 31 | 141 | 0.929 |
| **A*2902** | 0 | 16 | 158 | 0.0460 |  | 0 | 15 | 158 | 1.000 |
| **A*3001** | 0 | 16 | 158 | 0.0460 |  | 0 | 15 | 158 | 1.000 |
| **A*3002** | 3 | 36 | 135 | 0.1207 |  | 3 | 37 | 135 | 1.000 |
| **A*3402** | 1 | 8 | 165 | 0.0287 |  | 0 | 10 | 164 | 0.550 |
| **A*3601** | 2 | 26 | 146 | 0.0862 |  | 1 | 27 | 145 | 1.000 |
| **A*6601** | 0 | 8 | 166 | 0.0230 |  | 0 | 8 | 166 | 1.000 |
| **A*6802** | 7 | 33 | 134 | 0.1351 |  | 3 | 41 | 130 | 0.286 |
| **A*7401** | 1 | 31 | 142 | 0.0948 |  | 2 | 30 | 143 | 1.000 |
| **B*0702** | 3 | 18 | 153 | 0.0690 |  | 1 | 22 | 151 | 0.520 |
| **B*0801** | 0 | 6 | 168 | 0.0172 |  | 0 | 6 | 168 | 1.000 |
| **B*1302** | 0 | 4 | 170 | 0.0115 |  | 0 | 4 | 170 | 1.000 |
| **B*1402** | 0 | 7 | 167 | 0.0201 |  | 0 | 7 | 167 | 1.000 |
| **B*1503** | 4 | 23 | 147 | 0.0891 |  | 1 | 28 | 144 | 0.311 |
| **B*1510** | 0 | 25 | 149 | 0.0718 |  | 1 | 23 | 150 | 0.877 |
| **B*1516** | 0 | 1 | 173 | 0.0029 |  | 0 | 1 | 173 | 1.000 |
| **B*1801** | 0 | 7 | 167 | 0.0201 |  | 0 | 7 | 167 | 1.000 |
| **B*2703** | 0 | 0 | 0 | 0.0000 |  | 0 | 0 | 0 | NA |
| **B*3501** | 0 | 6 | 168 | 0.0172 |  | 0 | 6 | 168 | 1.000 |
| **B*4101** | 0 | 2 | 172 | 0.0057 |  | 0 | 2 | 172 | 1.000 |
| **B*4201** | 0 | 17 | 157 | 0.0489 |  | 0 | 16 | 157 | 1.000 |
| **B*4202** | 0 | 0 | 0 | 0.0000 |  | 0 | 0 | 0 | NA |
| **B*4403** | 0 | 5 | 169 | 0.0144 |  | 0 | 5 | 169 | 1.000 |
| **B*4501** | 2 | 18 | 154 | 0.0632 |  | 1 | 21 | 153 | 0.800 |
| **B*4901** | 0 | 10 | 164 | 0.0287 |  | 0 | 10 | 164 | 1.000 |
| **B*5101** | 0 | 1 | 173 | 0.0029 |  | 0 | 1 | 173 | 1.000 |
| **B*5301** | 2 | 34 | 138 | 0.1092 |  | 2 | 34 | 138 | 1.000 |
| **B*5701** | 0 | 0 | 0 | 0.0000 |  | 0 | 0 | 0 | NA |
| **B*5703** | 1 | 10 | 163 | 0.0345 |  | 0 | 12 | 162 | 0.826 |
| **B*5801** | 1 | 11 | 162 | 0.0374 |  | 0 | 13 | 161 | 0.833 |
| **B*5802** | 4 | 23 | 147 | 0.0891 |  | 1 | 28 | 144 | 0.310 |
| **B*8101** | 2 | 15 | 157 | 0.0546 |  | 1 | 18 | 156 | 0.786 |
| **Cw*0210** | 2 | 27 | 145 | 0.0891 |  | 1 | 28 | 144 | 1.000 |
| **Cw*0302** | 0 | 1 | 173 | 0.0029 |  | 0 | 1 | 173 | 1.000 |
| **Cw*0304** | 0 | 22 | 152 | 0.0632 |  | 1 | 21 | 153 | 1.000 |
| **Cw*0401** | 13 | 52 | 109 | 0.2241 |  | 9 | 61 | 105 | 0.498 |
| **Cw*0602** | 4 | 42 | 128 | 0.1437 |  | 4 | 43 | 128 | 1.000 |
| **Cw*0701** | 2 | 37 | 135 | 0.1178 |  | 2 | 36 | 135 | 1.000 |
| **Cw*0702** | 0 | 9 | 165 | 0.0259 |  | 0 | 9 | 165 | 1.000 |
| **Cw*0704** | 1 | 4 | 169 | 0.0172 |  | 0 | 6 | 168 | 0.750 |
| **Cw*0802** | 1 | 12 | 161 | 0.0402 |  | 0 | 13 | 160 | 1.000 |
| **Cw*1601** | 0 | 25 | 149 | 0.0718 |  | 1 | 23 | 150 | 0.877 |
| **Cw*1701** | 1 | 27 | 146 | 0.0833 |  | 1 | 27 | 146 | 1.000 |
| **Cw*1801** | 1 | 19 | 154 | 0.0603 |  | 1 | 20 | 154 | 1.000 |

^a^ Expected counts based on the assumption of Hardy-Weinberg equilibrium

^b^ p-value for Fisher Freeman-Halton test (Freeman, G. H. and J. H. Halton. 1951. Note on an exact treatment of contingency, goodness of fit and other problems of significance. Biometrika 38:14–49); NA: not applicable.
