# Supplementary figures and images for: High-Throughput High-Resolution Class I HLA Genotyping in East Africa
Source: PLoS One. 2010 May 20;5(5):e10751. doi: 10.1371/journal.pone.0010751 (PMC2873994; doi:10.1371/journal.pone.0010751)

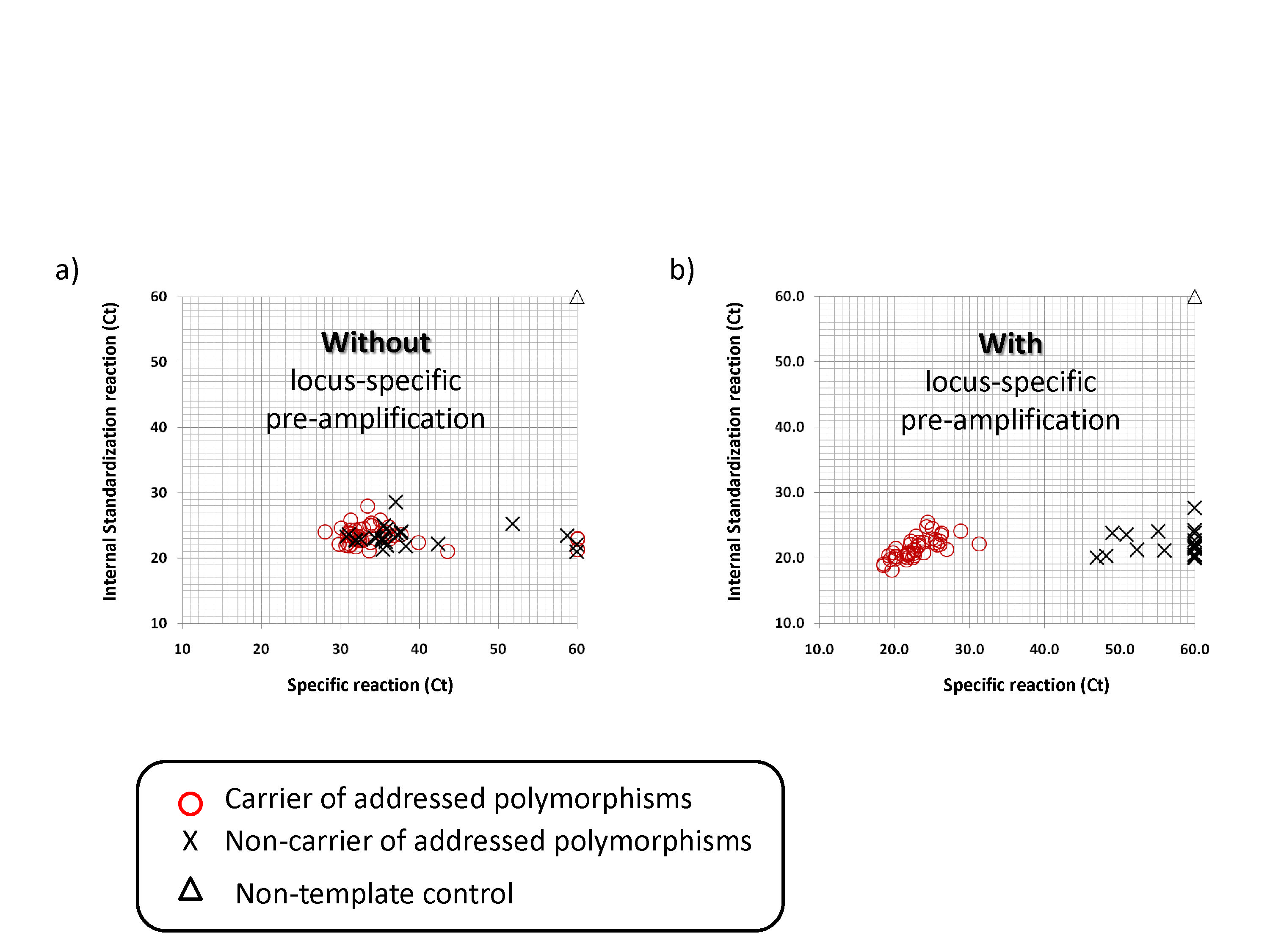

Supplement: Figure S1 — Locus-specific pre-amplification is necessary for optimal performance of SSP-real time PCR-based HLA typing. Performance of reaction HLA-B016 (which is specific for HLA-B*0702, HLA-B*0801, HLA-B*1402, HLA-B*1503, HLA-B*1510, HLA-B*1801, HLA-B*4201, HLA-B*4202, and HLA-B*8101) on Ugandan samples a) before, and b) after subjecting to HLA-B-specific PCR pre-amplification. Red circles and black crosses depict carriers and non-carriers of the addressed polymorphisms, respectively. Non-template controls are depicted by triangles. See text for details. (1.55 MB TIF) [file pone.0010751.s010.tif]

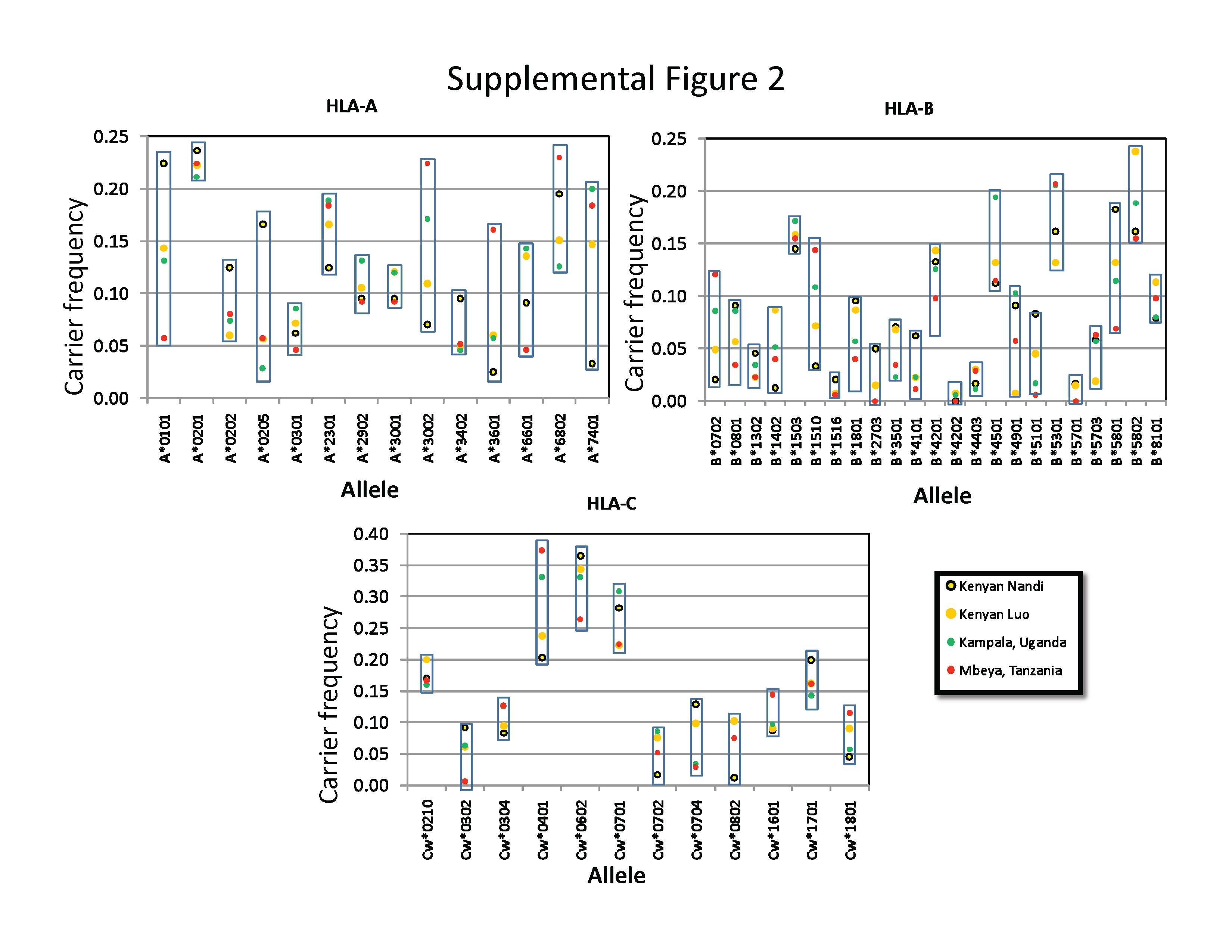

Supplement: Figure S2 — Carrier frequencies of major class I HLA alleles in East African populations. Carrier frequencies of alleles addressed by the current platform are shown for the Kenyan Highlander [Nandi] [32] (yellow), Kenyan Lowlander [Luo] (orange) [32], Uganda [33] (green) and the current Tanzanian population (red). (0.73 MB TIF) [file pone.0010751.s011.tif]

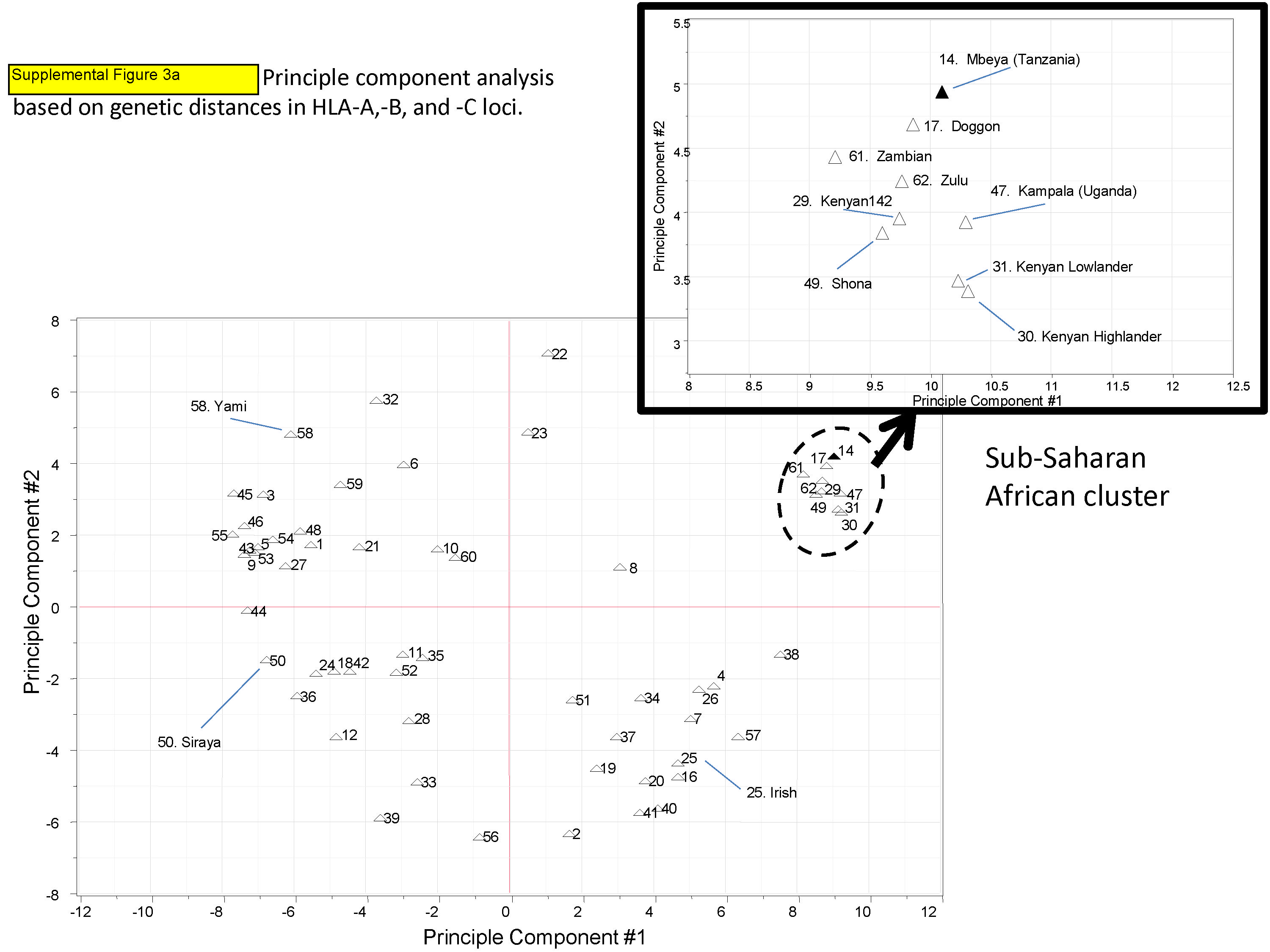

Supplement: Figure S3 — Principal component analysis showing the location of the Mbeya, Tanzania population in the context of global class I HLA genetic diversity. Principal component analysis (PCA) based on genetic distances in HLA-A,-B, and -C loci. Reference world populations were retrieved from the dbMHC [75]. In interest of clarity, only the first and second principal components are shown, which account for 79.4% of the variance. The composition of the outlined sub-Saharan cluster is shown in detail in the inset. The expanded box shows a close up the sub-Saharan populations. Populations are labeled as follows: 1: AmericanSamoa (American Samoa, United States), 2: Amerindian (United States), 3: Ami97 (Taiwan), 4: ArabDruze (Israel), 5: Atayal (Taiwan), 6: Bari (Venezuela), 7: Brazilian Admixed (Brazil), 8: Bulgarian (Bulgaria), 9: Bunun (Taiwan), 10: Canoncito (New Mexico, United States), 11: CapeYork (Australia), 12: Chinese (China), 14: Mbeya (Tanzania), 16: Czech (Czech Republic), 17: Doggon (Mali), 18: Filipino (Phillipines), 19: Finn90 (Finland), 20: Georgian (Georgia), 21: GrooteEylandt (Australia), 22: Guarani-Kaiowa (Brazil), 23: Guarani-Nandewa (Brazil), 24: Hakka (Taiwan), 25: Irish (Ireland), 26: IsraeliJews (Irish), 27: Ivatan (Philippines), 28: JavaneseIndonesian (Singapore), 29: Kenyan142 (Kenya), 30: KenyanHighlander (Kenya), 31: KenyanLowlander (Kenya), 32: Kimberley (Australia), 33: Korean200 (South Korea), 34: Kurdish (Georgia), 35: Malay (Singapore), 36: Minnan (Taiwan), 37: NewDelhi (India), 38: African American (United States), 39: Asian American (United States), 40: Caucasian (United States), 41: Hispanic (United States), 42: Okinawan (United States), 43: Paiwan51 (Taiwan), 44: Pazeh (Taiwan), 45: Puyuma49 (Taiwan), 46: Rukai (Taiwan), 47: Kampala, Uganda (Uganda), 48: Saisiat (Taiwan), 49: Shona (Zimbabwe), 50: Siraya (Taiwan), 51: Tamil (South Africa), 52: Thai (Singapore), 53: Thao (Taiwan), 54: Toroko (Taiwan), 55: Tsou (Taiwan), 56: Tuva (Taiwan), 58: Yami (Ta [file pone.0010751.s012.tif]

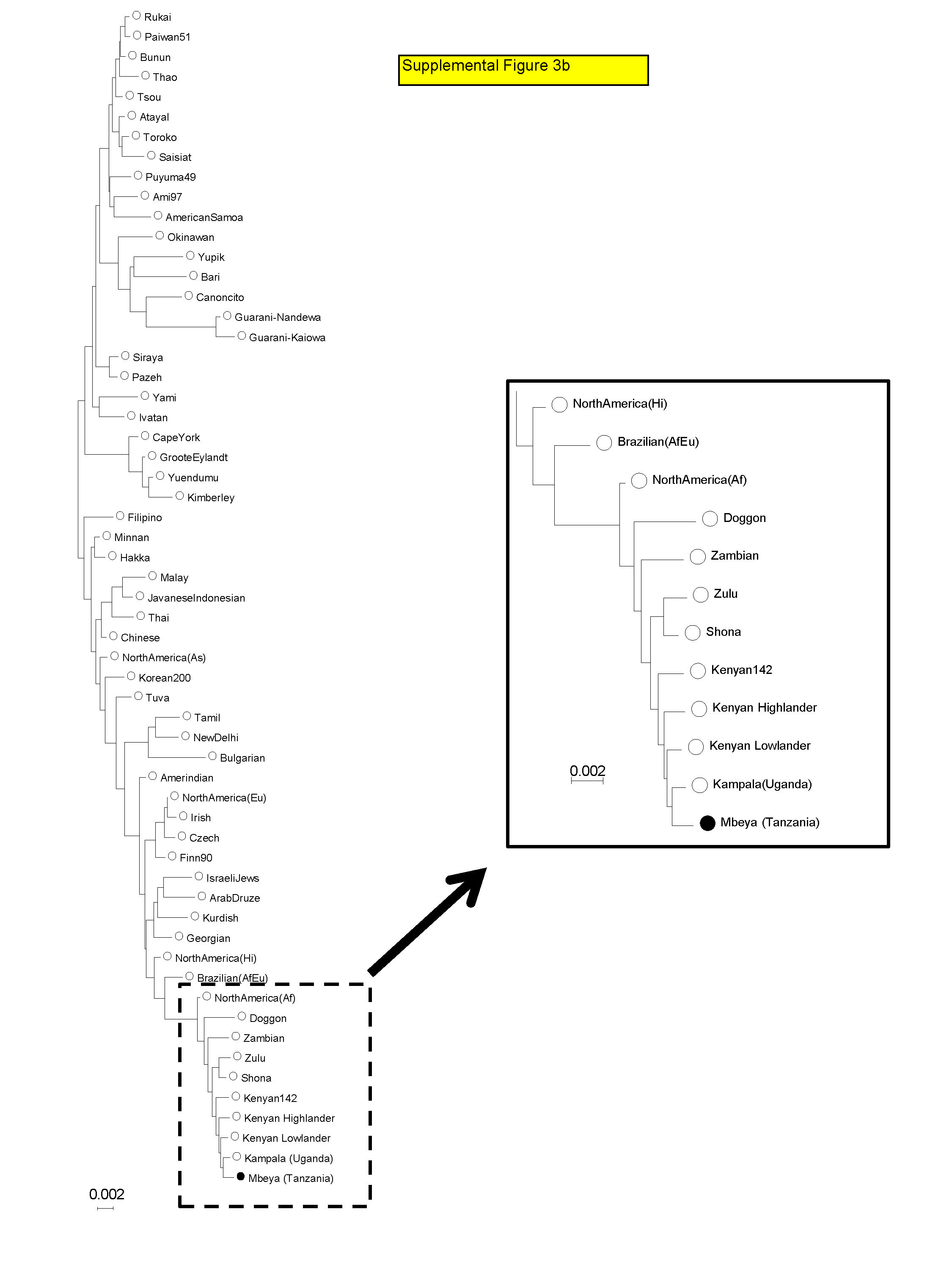

Supplement: Figure S4 — Dendrogram showing the location of the Mbeya, Tanzania population in the context of global class I HLA genetic diversity. The unrooted dendrogram was built using combined HLA-A,-B, -C allele frequency derived genetic distances. The expanded box shows a close up the sub-Saharan populations. Populations are labeled as follows: 1: AmericanSamoa (American Samoa, United States), 2: Amerindian (United States), 3: Ami97 (Taiwan), 4: ArabDruze (Israel), 5: Atayal (Taiwan), 6: Bari (Venezuela), 7: Brazilian Admixed (Brazil), 8: Bulgarian (Bulgaria), 9: Bunun (Taiwan), 10: Canoncito (New Mexico, United States), 11: CapeYork (Australia), 12: Chinese (China), 14: Mbeya (Tanzania), 16: Czech (Czech Republic), 17: Doggon (Mali), 18: Filipino (Phillipines), 19: Finn90 (Finland), 20: Georgian (Georgia), 21: GrooteEylandt (Australia), 22: Guarani-Kaiowa (Brazil), 23: Guarani-Nandewa (Brazil), 24: Hakka (Taiwan), 25: Irish (Ireland), 26: IsraeliJews (Irish), 27: Ivatan (Philippines), 28: JavaneseIndonesian (Singapore), 29: Kenyan142 (Kenya), 30: KenyanHighlander (Kenya), 31: KenyanLowlander (Kenya), 32: Kimberley (Australia), 33: Korean200 (South Korea), 34: Kurdish (Georgia), 35: Malay (Singapore), 36: Minnan (Taiwan), 37: NewDelhi (India), 38: African American (United States), 39: Asian American (United States), 40: Caucasian (United States), 41: Hispanic (United States), 42: Okinawan (United States), 43: Paiwan51 (Taiwan), 44: Pazeh (Taiwan), 45: Puyuma49 (Taiwan), 46: Rukai (Taiwan), 47: Kampala, Uganda (Uganda), 48: Saisiat (Taiwan), 49: Shona (Zimbabwe), 50: Siraya (Taiwan), 51: Tamil (South Africa), 52: Thai (Singapore), 53: Thao (Taiwan), 54: Toroko (Taiwan), 55: Tsou (Taiwan), 56: Tuva (Taiwan), 58: Yami (Taiwan), 59: Yuendumu (Australia), 60: Yupik (Alaska, United States), 61: Zambian (Zambia), 62: Zulu (South Africa). The Tanzanian population is depicted as a filled triangle. See text for details. (0.86 MB TIF) [file pone.0010751.s013.tif]
